# Physiological and protein profiling analysis provides insight into the underlying molecular mechanism of potato tuber development regulated by jasmonic acid *in vitro*

**DOI:** 10.1186/s12870-022-03852-x

**Published:** 2022-10-10

**Authors:** Jianlong Yuan, Lixiang Cheng, Huijun Li, Congcong An, Yuping Wang, Feng Zhang

**Affiliations:** 1 State Key Laboratory of Aridland Crop Science, Gansu Key Laboratory of Crop Improvement & Germplasm Enhancement, College of Agronomy, Gansu Agricultural University, Lanzhou, 730070 China; 2 College of Horticulture, Gansu Agricultural University, Lanzhou, China

**Keywords:** Potato, Differentially expressed proteins, Jasmonic acid, Proteome, Tuber development, iTRAQ

## Abstract

**Background:**

Jasmonates (JAs) are one of important phytohormones regulating potato tuber development. It is a complex process and the underlying molecular mechanism regulating tuber development by JAs is still limited. This study attempted to illuminate it through the potential proteomic dynamics information about tuber development *in vitro* regulated by exogenous JA.

**Results:**

A combined analysis of physiological and iTRAQ (isobaric tags for relative and absolute quantification)-based proteomic approach was performed in tuber development *in vitro* under exogenous JA treatments (0, 0.5, 5 and 50 μΜ). Physiological results indicated that low JA concentration (especially 5 μM) promoted tuber development, whereas higher JA concentration (50 μM) showed inhibition effect. A total of 257 differentially expressed proteins (DEPs) were identified by iTRAQ, which provided a comprehensive overview on the functional protein profile changes of tuber development regulated by JA. The Gene Ontology (GO) and Kyoto Encyclopedia of Genes and Genomes (KEGG) pathway enrichment analysis indicated that low JA concentration (especially 5 μM) exhibited the promotion effects on tuber development in various cellular processes. Some cell wall polysaccharide synthesis and cytoskeleton formation-related proteins were up-regulated by JA to promote tuber cell expansion. Some primary carbon metabolism-related enzymes were up-regulated by JA to provide sufficient metabolism intermediates and energy for tuber development. And, a large number of protein biosynthesis, degradation and assembly-related were up-regulated by JA to promote tuber protein biosynthesis and maintain strict protein quality control during tuber development.

**Conclusions:**

This study is the first to integrate physiological and proteomic data to provide useful information about the JA-signaling response mechanism of potato tuber development *in vitro*. The results revealed that the levels of a number of proteins involved in various cellular processes were regulated by JA during tuber development. The proposed hypothetical model would explain the interaction of these DEPs that associated with tuber development *in vitro* regulated by JA.

**Supplementary Information:**

The online version contains supplementary material available at 10.1186/s12870-022-03852-x.

## Background

Potato (*Solanum tuberosum* L.) is one of the most important food and vegetable crops worldwide. Potato tuber development is important for the formation of tuber yield and quality traits. Potato tuber development process includes two aspects: (a) the morphological development of tubers, and (b) the biochemical changes resulting in resource storage [1]. At the morphological aspect, potato tubers are derived from underground stems or stolons that undergo a series of development processes, including stolon formation and growth, tuberization induction, tuber initiation and growth [2]. The biochemical change process is accompanied by starch and storage protein accumulation in developing tubers, and coordinated by the expression of genes involved in the biosynthesis of these substances [2, 3]. Tuber formation and development is controlled by endogenous and external factors, which ensure that the time of tuber formation coincides with favorable developmental and environmental conditions [4]. Under favorable conditions, particularly short-day photoperiods and cool temperatures, some mobile signals such as FLOWERING LOCUS T (FT)-like protein (StSP6A) and StBEL5 mRNA originate in the leaf and then move to the stolon tip to regulate the onset of tuber formation [5–7]. Among these systems, the signaling and crosstalk of phytohormones play critical roles in tuberization [6, 8, 9].

Jasmonates (JAs) are lipid-derived phytohormones that regulate plant development and defense processes [10]. In potato, JAs have high tuber-inducing activity and promote tuber formation [11–14]. During the early stages of tuber formation, tuber initiation is associated with an increase of endogenous JA level [15]. JA stimulates tuber initiation and enlargement by antagonizing GA (Gibberellin), which exerts opposite actions in the cortical microtubule orientation to GA [8, 15]. The roles of JA in tuber formation mainly contribute to promoting radial cell expansion, meristem enlargement and early vascular tissue differentiation, thus facilitating the movement of substances to stolon tips [16, 17]. JA also stimulates the biosynthetic activity of plastid apparatus in the apical meristem cells of tubers for starch accumulation [18]. Further evidences for the involvement of JAs in potato tuberization have derived from affecting the expression of enzymes or proteins involved in JAs biosynthesis or signaling pathway. Lipoxygenases (LOXs) produce hydroperoxyl fatty acids that are precursors of JA and related compounds, which control potato tuber morphogenesis [19]. Suppression mutants produced by expressing antisense *POTLX-1* gene exhibit a significant reduction in the LOX activity of stolons and tubers, and a disruption of tuber formation [19]. Over-expression of JA carboxyl methyltransferase gene *JMT* in transgenic potato plants can enhance tuber yield and size as well as *in vitro* tuberization frequency [20]. A homologue of cytoplasmic/nuclear chitooligosaccharide-binding lectin might participate in JA-mediated signaling pathway involved in potato tuberization [21]. Overexpression of *StJAZ1-like* (a suppressor of JA signaling) negatively regulates tuber initiation by restricting the competence of tuber differentiation in stolon tips [22]. Despite these studies demonstrating the positive effects of JAs on tuberization, the information available on the molecular mechanism of tuber development regulated by JAs is still limited.

Proteomics approach as a powerful tool has identified some key proteins involved in potato tuber development [23–25]. Some phytohormone-responsive protein expression profiles of tuber development have also been reported in our recent research [26–28]. However, the information available on the protein expression profiles of tuber development regulated by JAs is still unknown. In the present study, the iTRAQ-based proteomic analysis was applied to investigate the molecular events of potato tuber development *in vitro* regulated by exogenous JA. This approach allows the simultaneous identification and quantitative comparison of peptides using tandem mass spectrometry (MS/MS) spectra. A comprehensive inventory of JA-responsive proteins involved in tuber development was established, which would provide new insights for the underlying molecular mechanism of potato tuber development regulated by JA.

## Materials and methods

### Plant materials, exogenous JA treatments and *in vitro* tuberization

*In vitro* plantlets of potato (*Solanum tuberosum* L. cv. Atlantic) as the experimental materials were propagated from single-node sections on the basal MS medium containing 3% (w/v) sucrose and 0.8% (w/v) agar (pH5.8), and cultured at 25 ± 2℃ under 16 h photoperiod (100 μmol m^−2^ s^−1^ light intensity) for four weeks [29]. The stolon induction from single-node explants and the tuber induction form stolon cuttings were described previously [28]. The tuber-induction MS medium was supplemented with different concentrations of exogenous JA (0, 0.5, 5 and 50 μΜ), respectively. After 40 days culture, the uniformly grown tubers were carefully harvested. The number of tubers per stolon was recorded from control and exogenous JA treatments, and the tuber size and weight were also measured. The fresh samples were used for physiological and biochemical analysis. The samples frozen in liquid nitrogen were stored at -80℃ for proteomic analysis.

### Starch, reducing sugar and sucrose content assay

Starch and sugars were extracted as described by Matsuura-Endo et al. [30]. Approximately 2 g of fresh tubers was homogenized and incubated with 20 mL 80% (v/v) ethanol at 70℃ for 3 h. After centrifugated at 10,000 g for 20 min, the supernatant was collected, vacuum-dried, dissolved in deionized water and passed through a membrane filter (0.2-μm, Millipore). The concentrations of fructose, glucose and sucrose in the filtrate were determined by HPLC (Model 1100 series, Agilent Technologies) with an Amide-80 column (HW-40F, TSKgel). Meanwhile, the pellet was vacuum-dried and added into 52% perchloric acid. The starch dissolved in perchloric acid was reacted with iodine solution, and then recorded the absorbance at 660 nm. The starch, reducing sugar and sucrose content were calculated on a fresh weight basis (mg g^−1^ FW).

### H_2_O_2_ content assay

H_2_O_2_ content was determined according to the method of Veljovic-Jovanovic et al. [31]. Approximately 0.5 g of fresh tubers was homogenized in liquid nitrogen with 2 mL of extraction solution [1 M HClO_4_ and 5% (w/v) polyvinyl pyrrolidone (PVP)]. The homogenate was centrifuged at 12,000 g for 10 min at 4℃. The supernatant was neutralized with 5 M K_2_CO_3_ in the presence of 100 mL 0.3 M phosphate buffer (pH5.6). The solution was then centrifuged at 12,000 g for 2 min at 4℃, and the sample was incubated for 10 min with 1 U ascorbate oxidase (Sigma). The reaction mixture consisted of 200 mL sample, 0.1 M phosphate buffer (pH6.5), 3.3 mM 3-dimethylaminobenzoic acid (DMAB) (Sigma), 0.07 mM 3-methyl, 2-benzo thiazolinone hydrazone (MBTH) (Sigma) and 0.3 U peroxidase (Sigma). The absorbance change was recorded at 590 nm, and the H_2_O_2_ content were calculated on a fresh weight basis (μM g^−1^ FW).

### Antioxidant enzyme activity assay

Approximately 2 g of fresh tubers was homogenized in liquid nitrogen with 10 mL of chilled extraction buffer [50 mM K-phosphate buffer (pH7.8), 1 mM Na-EDTA and 1% (w/v) PVP]. The homogenate was centrifuged at 15,000 g for 20 min at 4℃, and the supernatant was used for enzyme assay. All the steps in the preparation of enzyme extracts were performed at 4℃. Superoxide dismutase (SOD) activity was measured by nitroblue tetrazolium (NBT) method of Beyer and Fridovich and expressed as units mg^−1^ protein [32]. Ascorbate peroxidase (APX) activity was assayed by recording spectrophotometrically the decrease in ascorbate content at 290 nm (E = 2.47 mM^−1^ cm^−1^) according to the method of Ushimaru et al. [33] and expressed as units mg^−1^ protein. Catalase (CAT) activity was assayed by monitoring the consumption of H_2_O_2_ at 240 nm (E = 39.4 mM^−1^ cm^−1^) according to the method of Aebi [34] and expressed as units mg^−1^ protein.

### Protein extraction, digestion and iTRAQ labeling

Total tuber protein was extracted using an improved two-step precipitation method as described by Koistinen et al. [35]. Approximately 400 mg of frozen tuber samples were ground to a fine powder in liquid nitrogen. The powder was incubated with 1 mL of extraction buffer [50 mM Tris-HCl (pH8.0), 25 mM EDTA, 500 mM thiourea] containing 0.5% (v/v) β-mercaptoethanol for 30 min at 4 °C. Then, the homogenate was centrifuged at 13,000 g for 15 min at 4℃ to collect supernatants. The supernatants were mixed with Tris-phenol (pH8.0), and then centrifuged at 7,000 g for 10 min at 4 °C to collect phenol phase. The phenol phase was vortex mixed with five-fold volumes of 0.1 M cold ammonium acetate-methanol buffer and precipitated at -20 °C overnight. The samples were centrifuged at 12,000 g for 10 min at 4 °C to collect precipitation. Then, the pellet was washed with cold methanol, and centrifuged at 12,000 g for 10 min at 4 °C to collect precipitation. The wash step was repeated twice with acetone to remove methanol. The precipitation was air dried at room temperature for 5 min, and dissolved in lysis buffer [0.7 M urea, 2 M Thiourea, 2% (w/v) CHAPS, 2% (w/v) DTT] for 3 h. Finally, the samples were centrifuged at 12,000 g for 10 min at 4 °C to collect supernatants. Protein concentration was determined by BCA assay and stored at -80 °C. Protein digestion was performed according to the filter-aided sample preparation (FASP) procedure [36]. The digested peptides were labeled with iTRAQ Reagent-4 plex Multiplex Kit (SCIEX, USA). Four samples were labeled with iTRAQ tags 113 (0 μΜ JA), 114 (0.5 μΜ JA), 115 (5 μΜ JA) and 116 (50 μΜ JA) for three biological replicates. The labeled peptide mixtures were desalted by Agilent Zorbax Extend-C18 column and vacuum-dried.

### LC–MS/MS analysis

Each labeled peptide was dissolved with 2% acetonitrile (ACN) containing 0.1% formic acid (FA). The online chromatography separation was performed on an Eksigent nanoLC 415 system (SCIEX, USA) using ChromXP C_18_ column (3 μm, 75 μm × 15 cm, 120 A, ChromXP, Eksigent). The flow rate was 300 nL/min, and the linear gradient was 90 min (Mobile phase A: 2% ACN/0.1% FA, Mobile B: 95% ACN/0.1% FA). A Triple TOF 6600 tandem mass spectrometer (SCIEX, USA) was applied in MS analysis of the separated fractions. According to Zhu et al. [37], the data were acquired with a 2.4 kV ion spray voltage, 35 psi curtain gas, 12 psi nebulizer gas, and an interface heater temperature of 150 °C. The MS was scanned in IDA (Information-dependent acquisition) mode with a mass range 400-1,500 and an accumulation time of 250 ms. In each IDA cycle, 40 MS/MS spectra (80 ms, mass range 100-1,500) exceeding a threshold of 260 cps with a charge state of 2-4 were acquired. A rolling collision energy setting was applied to all precursor ions for collision-induced dissociation (CID), and the dynamic exclusion time was set for 16 s.

### Database searching and protein quantification

iTRAQ MS/MS data were analyzed using ProteinPilot software v.5.0 (SCIEX, USA) for protein identification and quantification [37]. Database searching for each sample were performed in the *Solanum tuberosum* database of Universal Protein Resource (UniProt). Only the proteins identified at FDR (false discovery rate) ≤ 0.01 and unique peptides ≥ 1 were considered for protein lists and further analysis. The detected protein threshold in the software was set to achieve 95% confidence. For protein quantification, the DEPs were identified on the basis of the ratios of differently labeled proteins. The proteomic analysis was performed with biological triplicates, and *t*-test was used to evaluate the significance of DEPs between groups at *p* < 0.05. Proteins with fold change > 1.2 or < 0.83-fold change and *p* < 0.05 were considered as significant DEPs.

### Bioinformatics analysis

The DEPs were functionally categorized according to GO annotation by BLAST2GO software [38]. The KEGG database (http://www.genome.jp/kegg/pathway.html) was employed to analyze the canonical biochemical pathways [39]. A hypergeometric test was used to find the significantly enriched GO terms and KEGG pathways (*p* < 0.05) of DEPs. Protein-protein interaction (PPI) networks were constructed by search against Interaction Genes/Proteins (STRING) database [40], and the interaction network was illustrated by Cytoscape software. The “factoextra”, “complexheatmap” and “vioplot” package in R language (Version 3.6.3) were used to generate the hierarchical clustering, heatmaps and violin plots of DEPs.

### Immunoblot analysis

Total protein (20 μg) was loaded on 10% TGX Stain-Free FastCast acrylamide gel electrophoresis (Bio-Rad, USA) at 80 V for 30 min and at 120 V for 70 min in a Mini-PROTEAN Tetra chamber (Bio-Rad, USA). Protein loading was determined with Chemidoc XRS system (Bio-Rad, USA). Subsequently, proteins were transferred to a 0.45 µm Immobilon-FL PVDF membrane (Merck Millipore, USA) at 70 V for 60 min, and sealed with PBST buffer (137 mM NaCl, 2.7 mM KCl, 10 mM Na_2_HPO_4_, 2 mM KH_2_PO_4_, 0.1% Tween 20) containing 5% (w/v) nonfat milk for 1 h. In order to save antibody and chromogenic reagent, we cut the membrane into strips using the molecular weight standard as a guide after transfer to a PVDF membrane. The primary antibodies (PhytoAb, USA) of HSP90, LOX2 and COI1 were used at 1:1000 dilutions for 1 h, and the secondary antibody HRP-linked Goat anti-mouse IgG (PhytoAb, USA) and HRP-linked Goat anti-rabbit IgG (PhytoAb, USA) were also used at 1:1000 dilutions for 1 h. Western Lightning™ Chemiluminescence Reagent Plus (Perkinelmer, USA) was adopted for color rendering, and the gel imaging system was employed for imaging. Protein expression was performed using Image Lab 6.1 software as the ratio of the tested proteins to total protein.

### Statistical analysis

Statistical analysis was carried out with three biological replicates for physiological and proteomic analyses. The repeated measurement was given as means ± standard error (SE). For the data of physiological analyses, the significant differences were analyzed by Duncan’s multiple range test (*p* < 0.05). For the data of proteomic analyses, *t*-test was used to evaluate the significance of DEPs between two groups, and one-way analysis of variance (ANOVA) followed by Bonferroni correction was performed for the multiple comparisons of DEPs. The *p*-values and corrected *p*-values (*P*_adj_) < 0.05 were considered to be statistically significant.

## Results

### Effects of exogenous JA on tuber tuberization *in vitro*

**Fig. 1:**
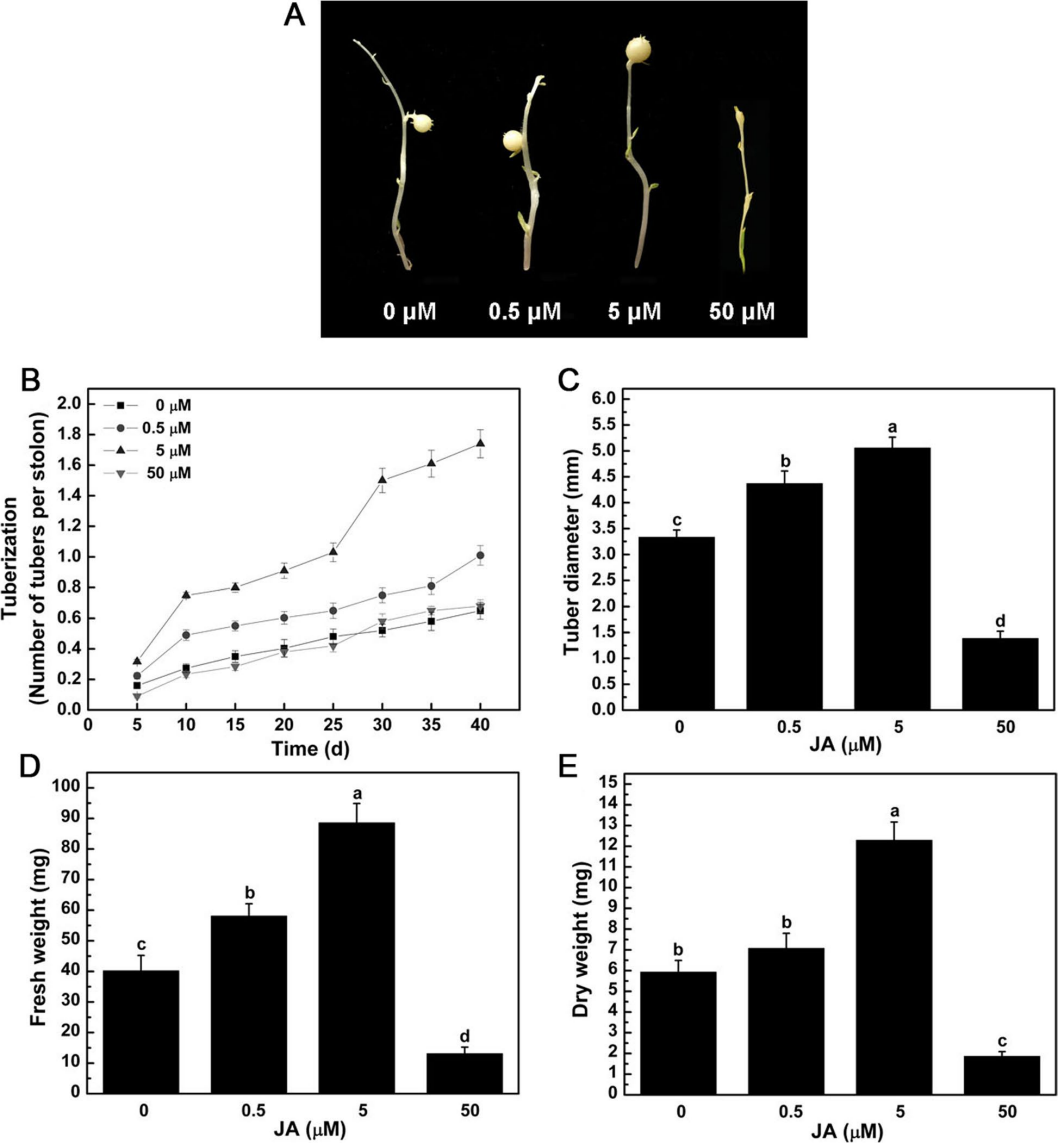
Effects of exogenous JA on tuber tuberization *in vitro*. **A** Tuber morphology, **B** Tuberization (number of tubers per stolon), **C** Tuber diameter, **D** Tuber fresh weight, **E** Tuber dry weigh. Data are presented as means ± SE for three independent experiments. Different letters indicate that the means differ significantly (*p* < 0.05) according to Duncan’s multiple range test

There were visible morphological changes of tubers *in vitro* treated by exogenous JA (Fig. 1). The low JA concentration (0.5 and 5 μM) promoted tuber development, whereas the high JA concentration (50 μM) showed inhibition effect. The tuber size was obviously increased under 5 μM JA treatment (Fig. 1A). Compared with control, the tuberization per stolon was significantly increased under 0.5 and 5 μM JA treatments, and there was no significant change under 50 μM JA treatment (Fig. 1B). At the end of culture period (40_th_ d), the tuberization per stolon of 0.5 and 5 μM JA treatments were 1.55 and 2.68-folds than that of control, respectively. The tuber diameter was significantly increased by 31.13% and 51.76% under 0.5 and 5 μM JA treatments, and decreased by 58.44% under 50 μM JA treatment (Fig. 1C), respectively. Similarly, the fresh weight of tubers was significantly increased by 44.46% and 120.31% under 0.5 and 5 μM JA treatments, and decreased by 67.42% under 50 μM JA treatment (Fig. 1D), respectively. Compared with control, there was no significant change in dry weight of tubers under 0.5 μM JA treatment, whereas it was significantly increased by 107.25% under 5 μM JA treatment, and decreased by 68.63% under 50 μM JA treatment (Fig. 1E).

### Physiological changes induced by exogenous JA of tubers *in vitro*

**Fig. 2:**
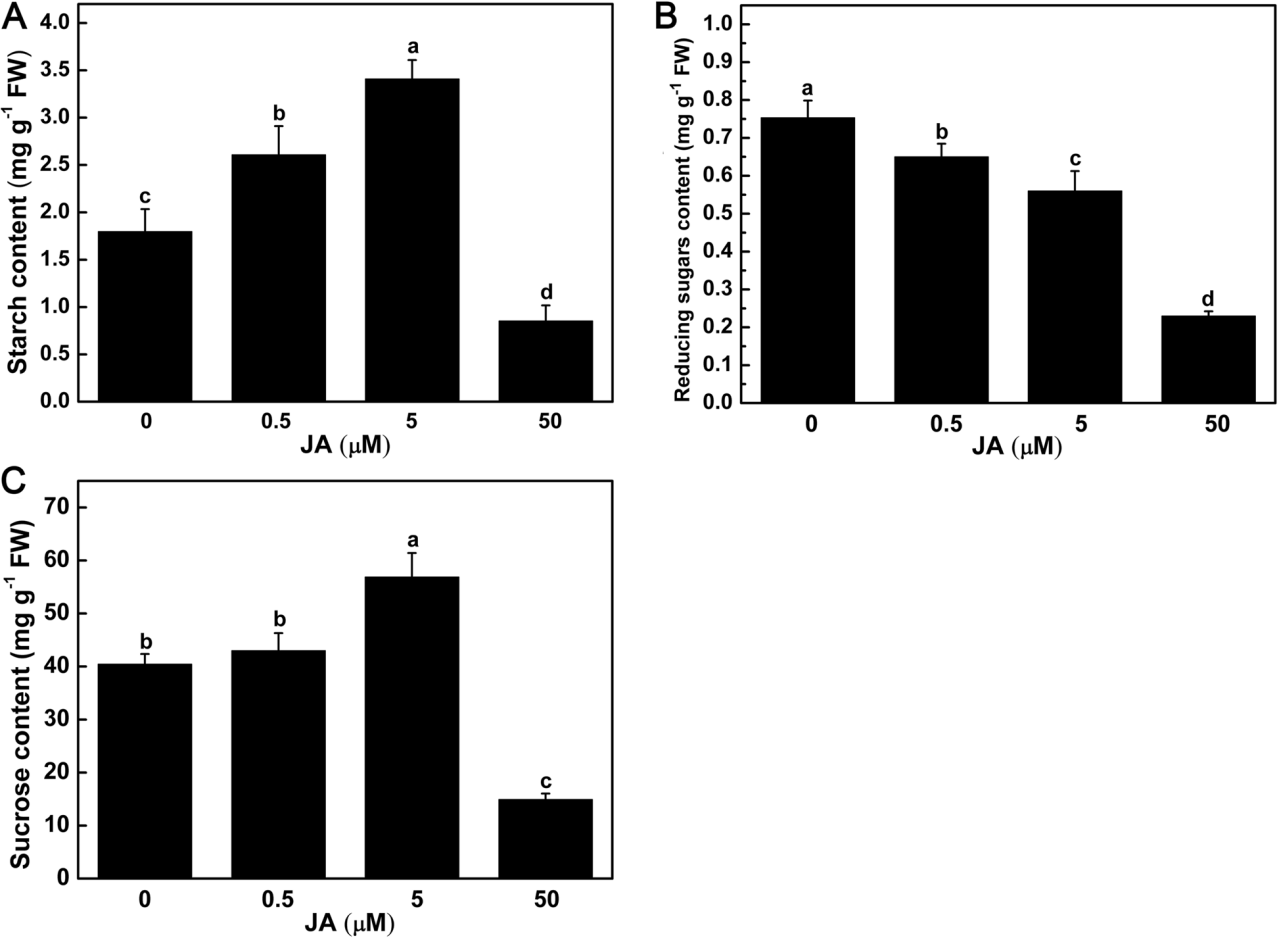
Effect of exogenous JA on the starch (**A**), reducing sugars (**B**) and sucrose (**C**) content of tubers *in vitro*. Data are presented as means ± SE for three independent experiments. Different letters indicate that the means differ significantly (*p* < 0.05) according to Duncan’s multiple range test

**Fig. 3:**
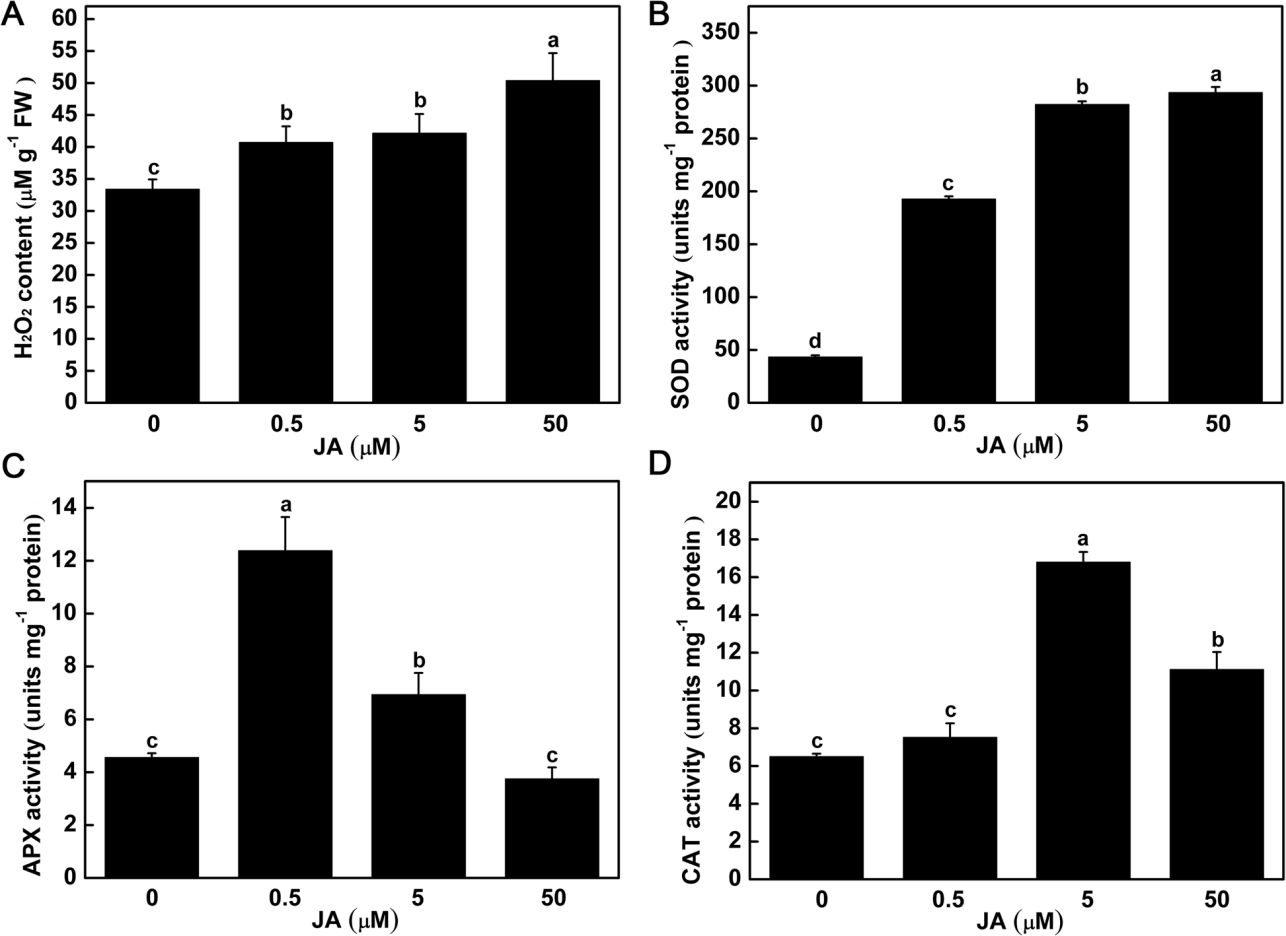
Effect of exogenous JA on the H_2_O_2_ content and antioxidant enzyme activity of tubers *in vitro*. After 40 days culture, the H_2_O_2_ content (**A**), SOD activity (**B**), APX activity (**C**) and CAT activity (**D**) of tubers were measured. Data are presented as means ± SE for three independent experiments. Different letters indicate that the means differ significantly (*p* < 0.05) according to Duncan’s multiple range test

Exogenous JA caused a series of physiological changes of tubers *in vitro*. The starch content was significantly increased by 45.12% and 89.62% under 0.5 and 5 μM JA treatments, and then obviously decreased under 50 μM JA treatment (Fig. 2A). With the increase of exogenous JA concentration, the reducing sugars content was significantly reduced by 13.72%, 25.66% and 69.47% under 0.5, 5 and 50 μM JA treatments (Fig. 2B), respectively. Compared with control, there was no significant change in sucrose content under 0.5 μM JA treatment, whereas it was significantly increased by 40.67% under 5 μM JA treatment, and decreased by 63.08% under 50 μM JA treatment (Fig. 2C). The activity of several antioxidant enzymes also showed obvious changes in tubers. With the increase of exogenous JA concentration, the H_2_O_2_ content was significantly increased by 21.89%, 26.23% and 49.70% under 0.5, 5 and 50 μM JA treatments (Fig. 3A), respectively. The SOD activity was also significantly increased with the increase of exogenous JA concentration (Fig. 3B). The APX activity was significantly increased under 0.5 and 5 μM JA treatments (Fig. 3C). The CAT activity was significantly increased under 5 and 50 μM JA treatments (Fig. 3D).

### Quantitative identification of JA-responsive DEPs with iTRAQ

A total of 2341 proteins were identified using iTRAQ analysis, and 2277 proteins were quantitatively analysed (Additional file 1: Table S1). According to Peptide FDR (false discovery rate) ≤ 0.01 and unique peptide number of protein ≥ 1, a total of 1157 proteins appearing simultaneously in three biological replicates were considered as the finally identified credible proteins (Additional file 2: Table S2). On the basis of the finally identified credible proteins, a total of 257 DEPs induced by JA during tuber development were determined according to fold change ≥ 1.2 or ≤ 0.83 and *p* < 0.05. Among these DEPs, 160 DEPs were identified between 0.5 μM JA treatments and control, including 21 up- and 139 down-regulated proteins (Additional file 3: Table S3-1). One hundred and fifteen DEPs were identified between 5 μM JA treatments and control, including 77 up- and 38 down-regulated proteins (Additional file 3: Table S3-2). Two hundred and ten DEPs were identified between 50 μM JA treatments and control, including 73 up- and 137 down-regulated proteins (Additional file 3: Table S3-3). One hundred and ninety-two DEPs were identified between 5 and 0.5 μM JA treatments, including 171 up- and 21 down-regulated proteins (Additional file 3: Table S3-4). One hundred and thirty-six DEPs were identified between 50 and 0.5 μM JA treatments, including 96 up- and 40 down-regulated proteins (Additional file 3: Table S3-5). One hundred and seventy-two DEPs were identified between 50 and 5 μM JA treatments, including 47 up- and 125 down-regulated proteins (Additional file 3: Table S3-6).

**Fig. 4:**
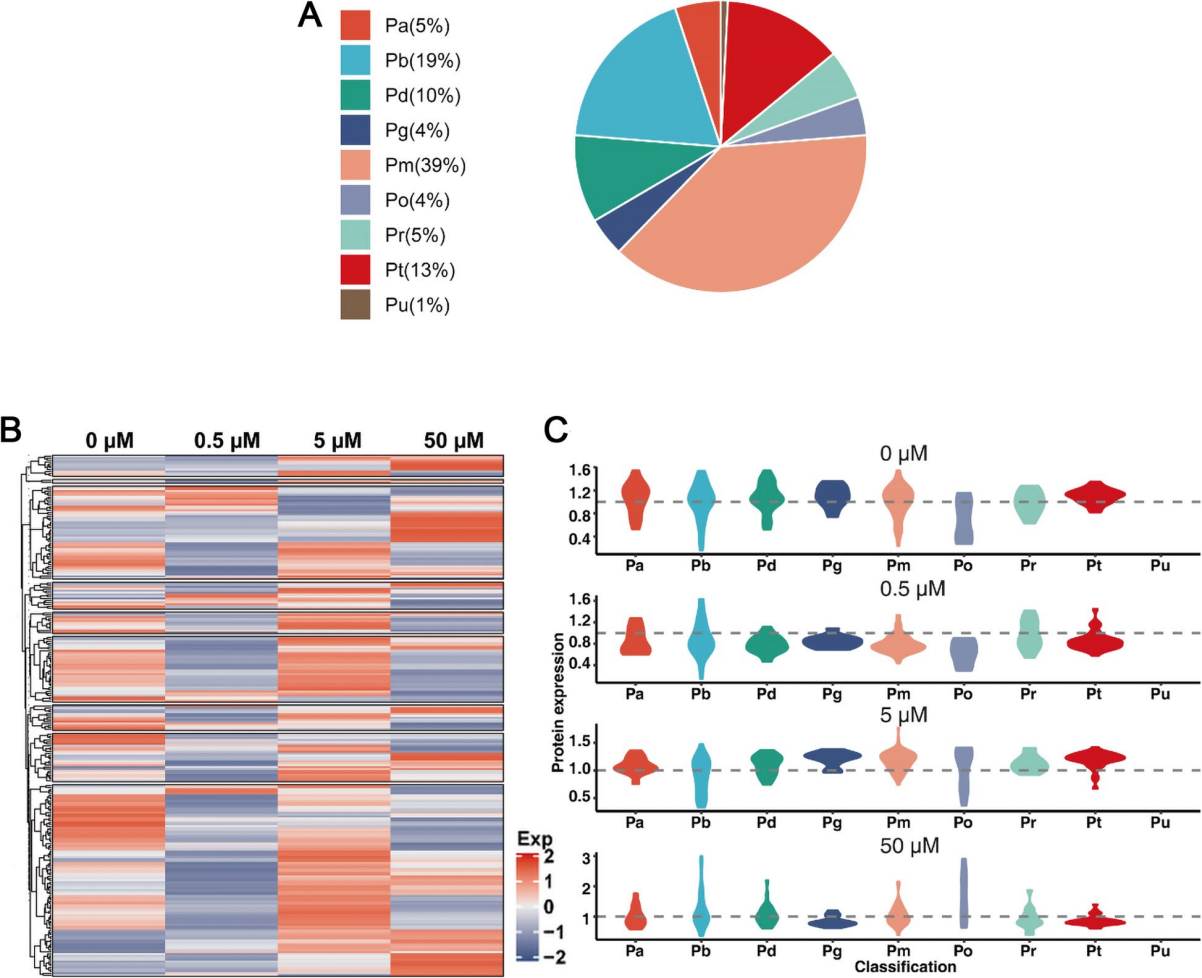
Functional classification, cluster analysis and distribution density of JA-responsive DEPs during tuber development *in vitro*. **A** The pie chart showed nine protein function categories of DEPs. **B** A heat-map displayed the differential expression patterns of DEPs in different functional categories. All the protein names and information of heatmap were listed in the Additional file 4: Table S4. **C** A violin plot displayed the protein expression distribution density of DEPs in different functional categories. The gray dotted line was the average value of protein expression. Pm, metabolism; Pb, protein biosynthesis and degradation; Pt, transcription and translation; Pd, cellular defense; Pg, signaling; Pr, transport, Pa, cell cycle and structure; Po, miscellaneous; Pu, unknown

Furthermore, the 257 DEPs were functionally classified according to Uniprot, KEGG and eggNOG database information (Fig. 4A). They were divided into nine function categories, including metabolism (Pm, 39%), protein biosynthesis and degradation (Pb, 19%), transcription and translation (Pt, 13%), cellular defense (Pd, 10%), cell cycle and structure (Pa, 5%), transport (Pr, 5%), signaling (Pg, 4%), miscellaneous (Po, 4%) and unknown (Pu, 1%). Furthermore, the differential expression patterns of these proteins in different functional categories were analyzed (Fig. 4B), and all the protein names and information of heatmap were listed in the Additional file 4: Table S4. Under 0.5 μM JA treatment, most of DEPs in different functional categories were down-regulated. Under 5 μM JA treatment, the up-regulated proteins were more than down-regulated proteins in different functional categories except for protein synthesis and degradation-related proteins (Pb). Under 50 μM JA treatment, most of the transcription and translation-related proteins (Pt) and signaling-related proteins (Pg) were down-regulated, whereas the protein biosynthesis and degradation-related proteins (Pb) were up-regulated. Additionally, the distribution patterns of DEP expression levels under different JA concentrations were also investigated (Fig. 4C). The expression distribution of cellular defense-related proteins (Pd), signaling-related proteins (Pg), metabolism-related proteins (Pm) and transcription and translation-related proteins (Pt) were relatively concentrated, which were mainly up-regulated under 5 μM JA and down-regulated under 0.5 and 50 μM JA treatment. The expression distribution of cell cycle and structure-related proteins (Pa) and transport-related proteins (Pr) were mainly up-regulated under 5 μM JA treatment. The expression distribution of protein biosynthesis and degradation-related proteins (Pb) and miscellaneous proteins (Po) were relatively dispersed under different JA concentrations.

### Cluster analysis of JA-responsive DEPs during tuber development

**Fig. 5:**
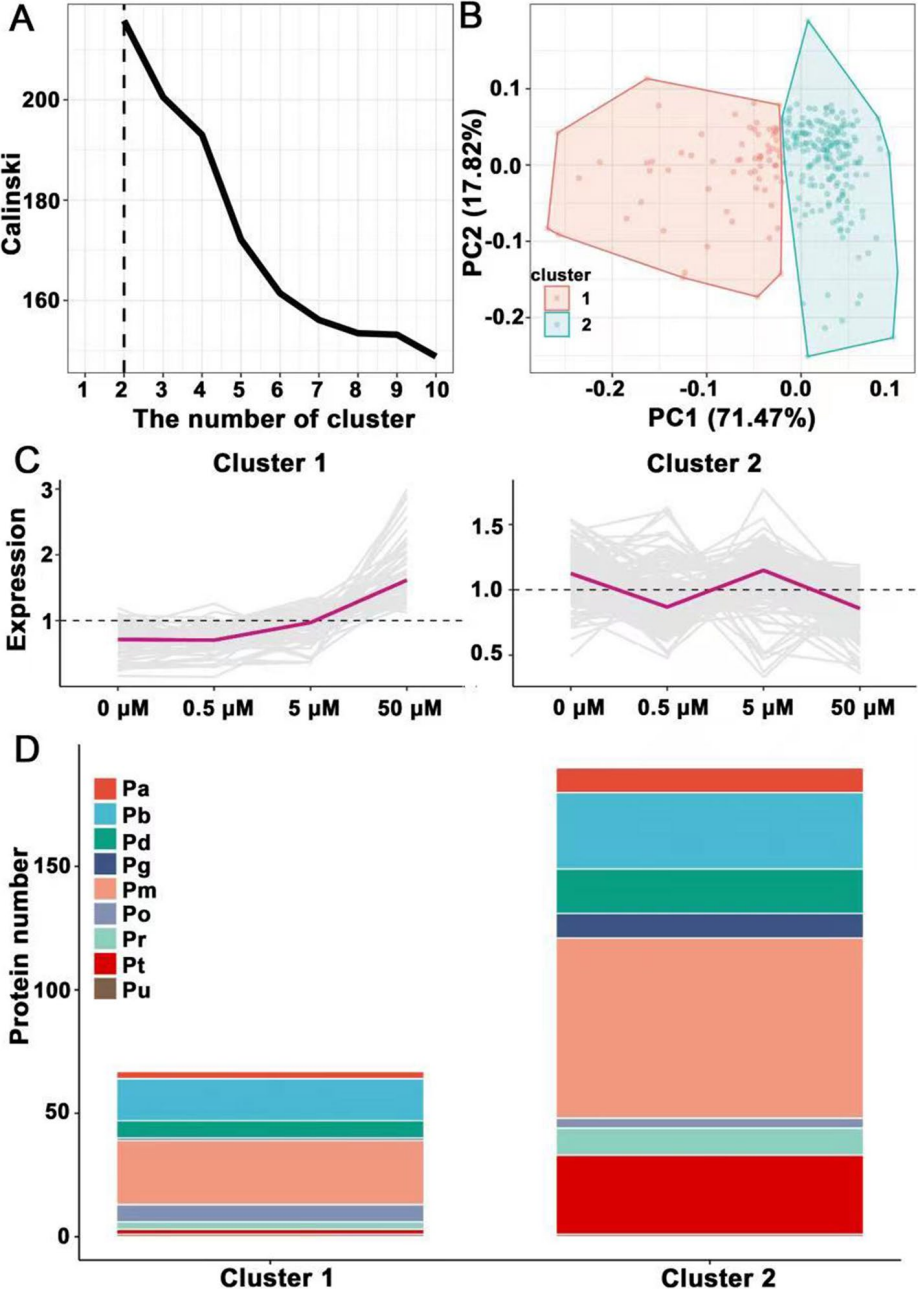
The expression pattern of JA-responsive DEPs in different function categories. **A** The optimal number of clusters was chosen by maximizing the Calinski-Harabasz index (Calinski) of DEPs. The black dashed line indicates the best number of K-median clusters. **B** The principal component analysis of DEPs distribution in two clusters. The red area represents cluster 1, and the blue area represents cluster 2. **C** All the DEPs were grouped into two clusters based on the similar expression pattern according to K-median clustering. **D** The function categories of DEPs in each cluster with similar expression pattern. Pm, metabolism; Pb, protein biosynthesis and degradation; Pt, transcription and translation; Pd, cellular defense; Pg, signaling; Pr, transport, Pa, cell cycle and structure; Po, miscellaneous; Pu, unknown

A K-median clustering analysis was applied to JA-responsive DEPs during tuber development (Fig. 5). The optimal number of clusters was chosen by maximizing the Calinski-Harabasz index (Calinski) of DEPs, which indicated that it was best to be divided into two K-median clusters (Fig. 5A). Furthermore, the principal component analysis (PCA) showed that the distribution of DEPs in both two clusters was concentrated, and the concentration degree of cluster 2 is better than that of cluster 1 (Fig. 5B). In cluster 1, the expression trend of DEPs showed a gradual up-regulation with the increase of exogenous JA concentration (Fig. 5C). The protein functions contained in cluster 1 were listed by the descending order of protein number as follows: metabolism (Pm), protein biosynthesis and degradation (Pb), cellular defense (Pd), miscellaneous (Po), cell cycle and structure (Pa), transport (Pr), transcription and translation (Pt), signaling (Pg) and unknown (Pu) (Fig. 5D). Cluster 2 was the most abundant group, and the expression trend of DEPs showed a down-regulation under 0.5 and 50 μM JA treatments and an up-regulation under 5 μM JA treatment (Fig. 5C). The protein functions contained in cluster 2 were listed by the descending order of protein number as follows: metabolism (Pm), protein biosynthesis and degradation (Pb), transcription and translation (Pt), cell cycle and structure (Pa), cellular defense (Pd), transport (Pr), signaling (Pg), miscellaneous (Po) and unknown (Pu) (Fig. 5D).

### Functional enrichment analysis of JA-responsive DEPs during tuber development

**Fig. 6:**
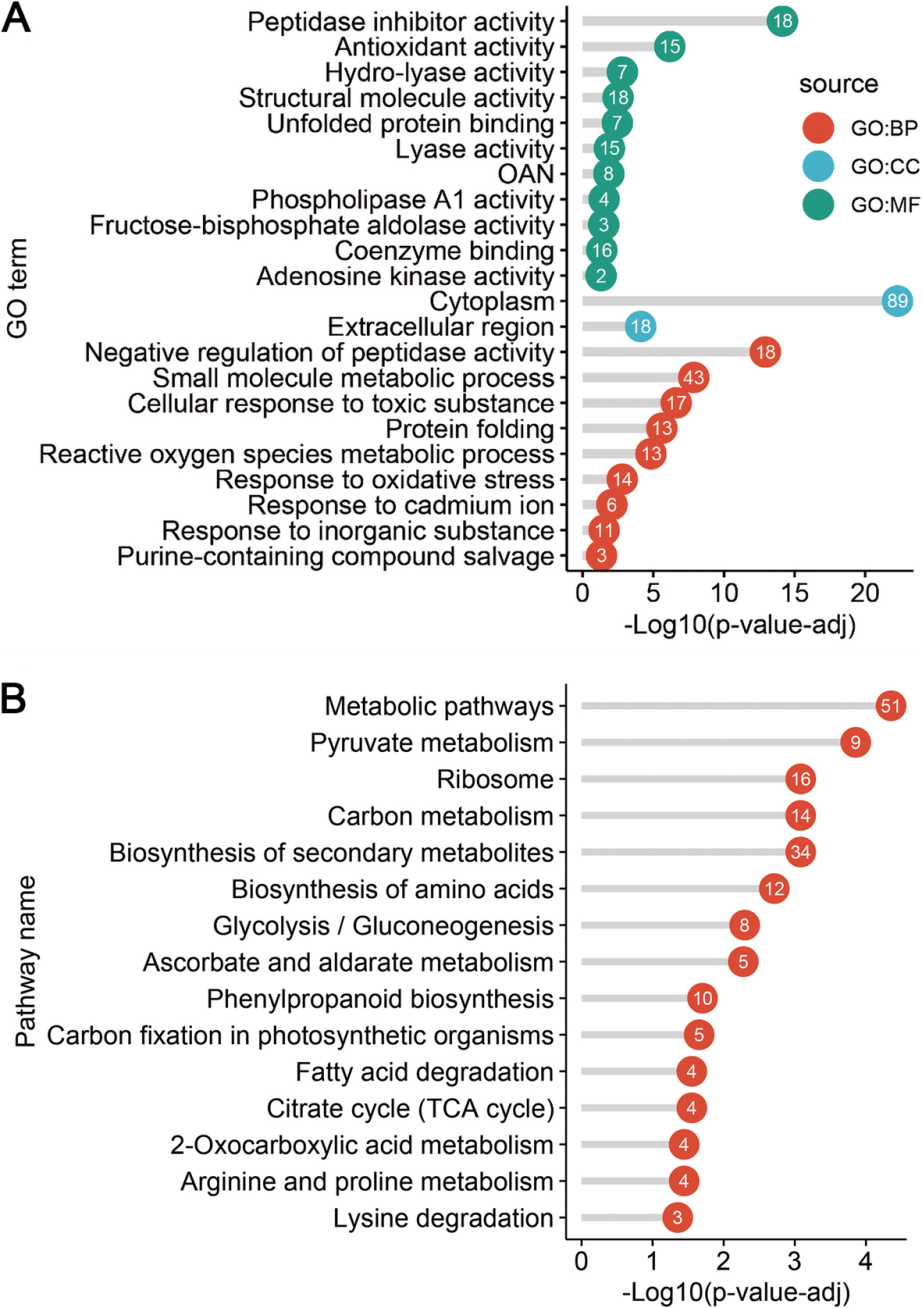
The GO term and KEGG pathway enrichment of JA-responsive DEPs during tuber development *in vitro*. **A** The GO categorization of DEPs. Three GO sources (BP, biological process; CC, cellular component; MF, molecular function) were represented by red, blue, and green spots, respectively. **B** The KEGG pathway annotation of DEPs. The number in the spot represents protein number

GO categorization and KEGG pathway annotation were used to display the functional enrichment of JA-responsive DEPs (Fig. 6). All the DEPs were categorized into three GO terms including biological process, cellular component and molecular function (Fig. 6A). In total, 22 GO categories were significantly enriched in three levels, including 11 GO terms in biological process, 2 GO terms in cellular component and 9 GO terms in molecular function. In terms of biological processes, the top five enriched GO terms were Negative regulation of peptidase activity (GO:0010466), Small molecule metabolic process (GO:0044281), Cellular response to toxic substance (GO:0097237), Protein folding (GO:0006457) and Reactive oxygen species metabolic process (GO:0072593), respectively. In terms of cell composition, the enriched GO terms were Cytoplasm (GO:0005737) and Extracellular region (GO:0005576), respectively. In terms of molecular function, the top five enriched GO terms were Peptidase inhibitor activity (GO:0030414), Antioxidant activity (GO:0016209), Hydro-lyase activity (GO:0016836), Structural molecule activity (GO:0005198) and Unfolded protein binding (GO:0051082), respectively. The KEGG pathway enrichment analysis indicated that the JA-responsive DEPs during tuber development were mainly enriched in 15 pathways (Fig. 6B). The top five enriched pathways were mainly focus on “Metabolic pathways”, “Pyruvate metabolism”, “Ribosome”, “Carbon metabolism” and “Biosynthesis of secondary metabolites”.

### Protein–protein interaction analysis of JA-responsive DEPs during tuber development

**Fig. 7:**
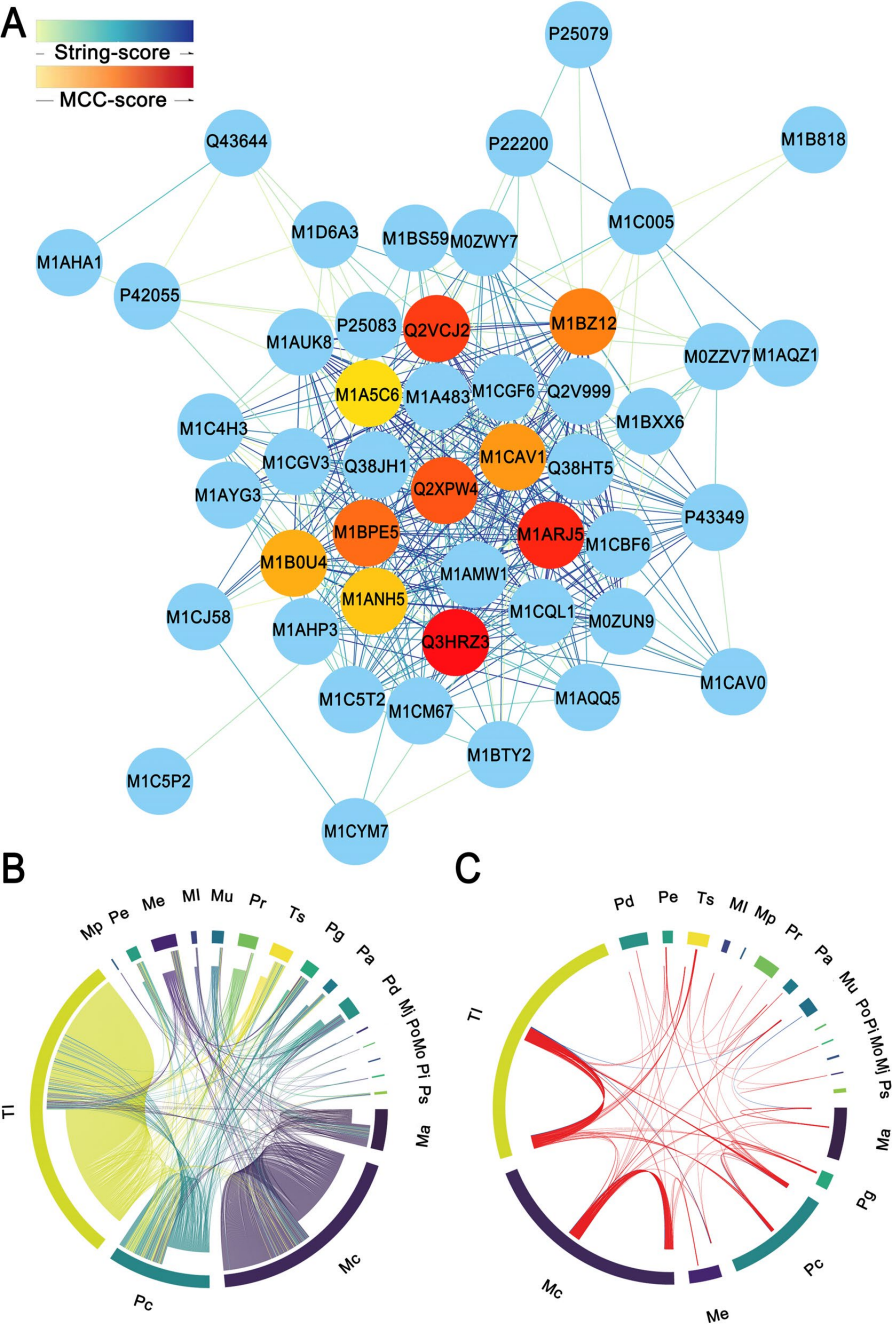
Interaction network of JA-responsive DEPs during tuber development *in vitro*. **A** The key node proteins in the interaction network were analyzed by Maximal Clique Centrality (MCC) algorithm. The ascending order STRING score is colored from light green to dark blue. The top ten proteins with MCC scores are sorted from lowest to highest and colored from yellow to red. **B** The interaction of DEPs with different functions. The corresponding functional categories of interacting proteins are represented by the ribbons with different colors in the chord diagram. **C** The expression correlation of interacting proteins with different functions. The red line indicates a positive relation (*p* < 0.05), and the blue line indicates a negative relation (*p* < 0.05). Ts, transcription; Tl, translation; Mc, carbohydrate metabolism; Ma, amino acid metabolism; Me, energy metabolism; Ml, phospholipid; Mp, inorganic phosphate ion metabolism; Mj, JA metabolism; Mu, nucleotide metabolism; Mo, other metabolism; Pe, proteases and peptidases; Pc, protein folding; Pr, transport and channel; Pg, signaling; Pa, cellular structure; Pd, defense; Pi, protease inhibitors; Ps, storage; Po, other proteins

The JA-responsive DEPs were analyzed using the STRING online search tool to build an interaction network of DEPs (Fig. 7). The Maximal Clique Centrality (MCC) algorithm was used to analyze the key node proteins in the interaction network (Fig. 7A). The top ten key node proteins were 40S Ribosomal protein S15A-1 (Q3HRZ3), 60S Ribosomal protein L4/L1 (M1ARJ5), Ribosomal protein L3 (Q2VCJ2), 60S Ribosomal protein L7A (Q2XPW4), 40S Ribosomal protein S24 (M1BPE5), 60S Ribosomal protein L8 (M1BZ12), 60S Ribosomal protein L18 (M1CAV1), 60S Ribosomal protein L6 (M1B0U4), Glycoprotein (M1ANH5) and Ribosomal protein (M1A5C6), respectively. Furthermore, the functional categories of interacting proteins were analyzed (Fig. 7B). The corresponding functional categories of interacting proteins were represented by the ribbons with different colors in a chord diagram, which mainly involved in translation (Tl), transcription (Ts), carbohydrate metabolism (Mc), amino acid metabolism (Ma), energy metabolism (Me), phospholipid metabolism (Ml), inorganic phosphate ion metabolism (Mp), JA metabolism (Mj), nucleotide metabolism (Mu), other metabolism (Mo), proteases and peptidases (Pe), protein folding (Pc), transport and channel (Pr), signaling (Pg), cellular structure (Pa), defense (Pd), protease inhibitors (Pi), storage proteins (Ps) and other proteins (Po). Among them, the proteins with relatively high interaction frequency were mainly involved in translation (Tl), protein folding (Pc), transport and channel (Pr), signaling (Pg), defense (Pd), transcription (Ts) and energy metabolism (Me) (Fig. 7B). Additionally, we also separated and visualized the positive and negative correlations among these functional categories of interacting proteins using a chord diagram (Fig. 7C). The thickness of chords illustrated the relative contribution of individual correlation coefficients to the global correlation. The ribbons within the circle correspond to significant correlations with a *p* < 0.05, and the red and blue ribbons indicated positive and negative coefficients, respectively. The data showed that there existed a universal positive correlation almost among the interacting proteins with all the above functional categories. For example, the translation (Tl)-related proteins were strongly correlated positively with the proteins involved in transcription (Ts), protein folding (Pc) and signaling (Pg). The carbohydrate metabolism (Mc)-related proteins were strongly correlated positively with the proteins involved in nucleotide metabolism (Mu) and amino acid metabolism (Ma). Negative correlations were observed only between the interacting proteins involved in translation (Tl) and nucleotide metabolism (Mu), nucleotide metabolism (Mu) and amino acid metabolism (Ma), translation (Tl) and energy metabolism (Me).

### Immunoblot analysis of key proteins in JA biosynthesis and regulatory pathway during tuber development

**Fig. 8:**
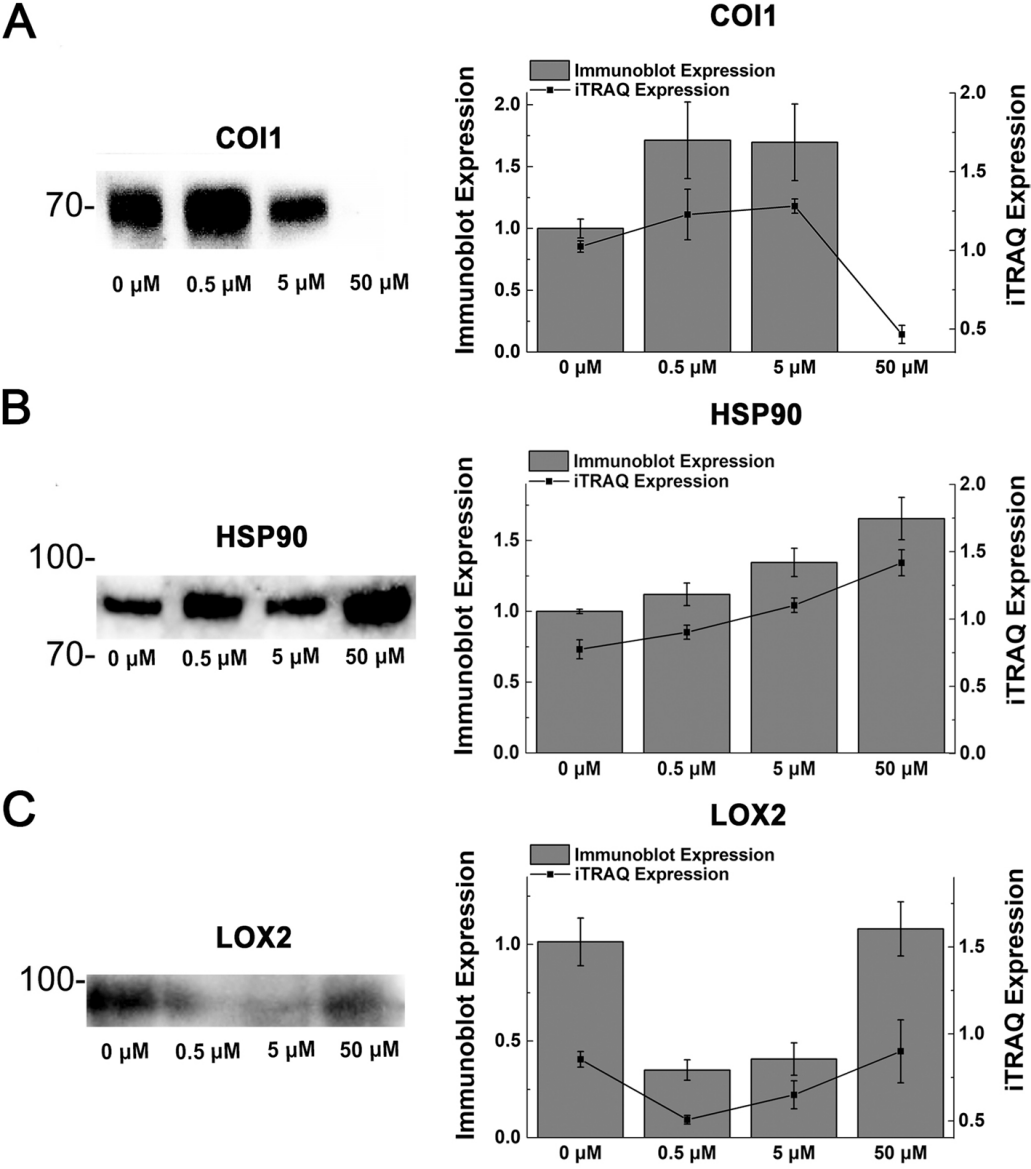
Immunoblot analysis of key proteins in JA biosynthesis and regulatory pathway during tuber development. The immunoblot expression and iTRAQ expression of COI1 (**A**), HSP90 (**B**), and LOX2 (**C**) were analyzed under different JA concentration. Data are presented as means ± SE for three independent experiments. In order to save antibody and chromogenic reagent, the membranes were cut into strips using the molecular weight standard as a guide after transfer to a PVDF membrane. The first membrane was cut just 1 cm above and below the 70 KDa molecular weight marker and used for COI1 immunoblot. The second membrane was cut just between the 70 KDa and 100 KDa molecular weight markers and used for HSP90 immunoblot. The third membrane was cut just 1 cm above and below the 100 KDa molecular weight marker and used for LOX2 immunoblot. The original gels and blot strips are presented in Additional files 5, 6, 7 and 8: Fig. S1-4

The expression patterns of Lipoxygenase 2 (LOX2), Coronatine insensitive 1 (COI1) and COI1/JAZ binding accessory protein (HSP90) in JA biosynthesis and regulatory pathway were determined during tuber development by immunoblot analysis (Fig. 8; Additional files 5, 6, 7 and 8: Fig. S1-4). Compared with control, the expression level of COI1 was increased by 71.3% and 69.7% under 0.5 and 5 μM JA treatments, and no expression was detected under 50 μM JA treatment (Fig. 8A). The expression level of HSP90 was increased by 12.0%, 34.5% and 65.5% under 0.5, 0.5 and 5 μM JA treatment than that of control (Fig. 8B). The expression level of LOX2 was reduced by 61.8% and 59.3% under 0.5 and 5 μM JA treatment than that of control, whereas slightly increased under 50 μM JA treatment (Fig. 8C). The immunoblot analysis results of LOX2 and HSP90 were consistent with proteome results.

## Discussion

Potato tuber development is a complex biological process that requires the coordinated interaction of endogenous and external factors [4]. Phytohormones play crucial roles in regulating morphological events of potato development [8]. It is well known that JAs are a kind of phytohormones with multiple functions in plants [10]. JAs are mainly involved in pathogen defense in many higher plants, whereas JAs has a specific role of tuber-inducing activities in potato [11, 14]. Numerous studies have focused on the favorable effects of JAs on tuber development [14, 17, 18]. However, the underlying molecular mechanism of tuber development regulated by JAs is still largely unknown. The present study attempted to integrate physiological and proteomic approach to illuminate the JA-signaling response mechanism of potato tuber development *in vitro*.

### The cell wall and cytoskeleton composition were regulated by JA to promote tuber cell expansion

**Fig. 9:**
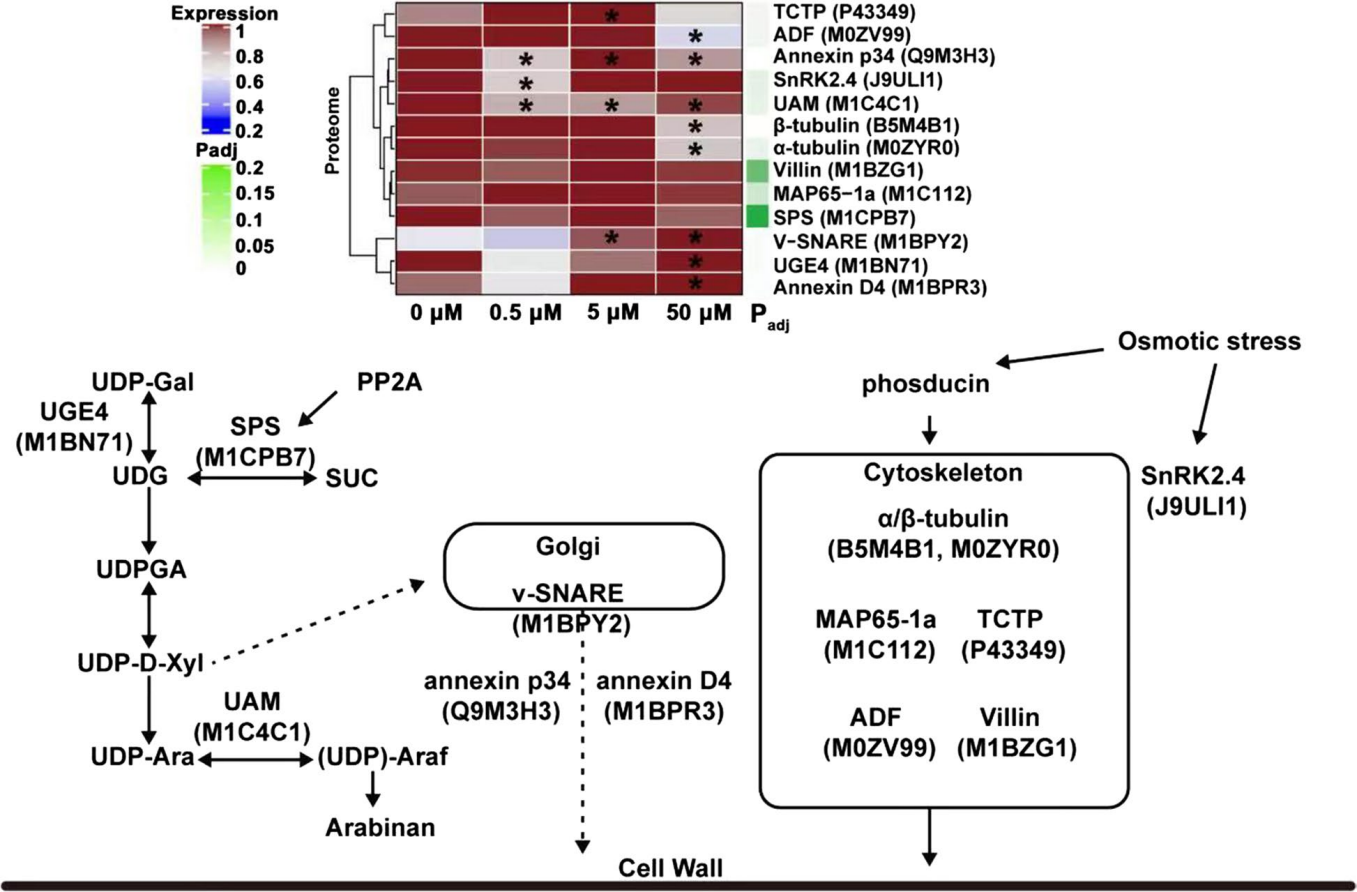
The JA-responsive DEPs involved in cell wall and cytoskeleton composition during tuber development *in vitro*. The heat-map presented the expression change of these DEPs. The significance of *t*-test was presented by “*” (*p* < 0.05). The green band indicated the corrected *p*-value (P_adj_ < 0.05, one-way ANOVA analysis of variance followed by Bonferroni correction for multiple comparison) was mapped as an annotation of heatmap. ADF, actin depolymerizing factor; MAP65-1a, microtubule-associated protein 65-1a; SnRK2.4, SNF1-releted protein kinases 2.4; SPS, sucrose-phosphate synthase; TCTP, translationally-controlled tumor protein; UAM, alpha-1,4-glucan-protein synthase; UGE4, UDP-glucose 4-epimerase; V-SNARE, soluble N-ethylmaleimide-sensitive fusion protein attachment protein receptors

Both the cell division and cell enlargement contribute to potato tuber development [41]. The changes of cell wall, plasma membrane and cytoskeleton can alter cell morphology [42]. Previous study has shown that JA can induce cell wall remodeling by sensing the osmotic pressure changes of cells [43]. In the present study, some cell wall synthesis-related proteins were found to be regulated by JA during potato tuber development. It appeared that JA might regulate the cell wall polysaccharide synthesis and the raw material transportation of cell wall biosynthesis, so as to provide a material basis for tuber expansion (Fig. 9). In the plant cell wall polysaccharide synthesis pathway, UDP-glucose 4-epimerase (UGE4) catalyzes the transformation between UDP-glucose and UDP-galactose [44]. The inhibition of UGE4 expression can alter the arrangement of plant root cells [44]. The down-regulation of alpha-1,4-glucan-protein synthase (UAM) can reduce xylan content in cell wall, and the decrease of xylan content in cell wall lead to cell enlargement [45, 46]. In this study, the UAM (M1C4C1) was significantly down-regulated (*p* < 0.05) and UGE4 (M1BN71) was slightly down-regulated under 0.5 and 5 μM JA treatment, whereas they were both significantly up-regulated (*p* < 0.05) under 50 μM JA treatment. It appeared that low JA concentration might promote tuber cell enlargement by regulating the composition of polysaccharides in cell wall during tuber development. Annexin and Vesicle transport v-SNARE involved in vacuole secretion and Golgi transport can affect cell wall formation by regulating cell wall polysaccharide transport [47, 48]. JA can enhance *ANNEXIN* expression, thus inhibiting glucan synthase activity [49, 50]. When pathogens invaded, the increased JA level can induce v-SNARE expression to accelerate polysaccharide deposition in cell wall and enhance cell wall strength [51, 52]. The v-SNARE (M1BPY2) was significantly up-regulated (*p* < 0.05) under 5 μM JA treatment, and the Annexin D4 (M1BPR3) showed a slight up-regulation. It was suggested that JA might also promote cell wall remodeling by accelerating the transport of cell wall synthesis elements during tuber development.

In addition, plant cell enlargement is accompanied cytoskeleton composition change, mainly the change of microtubule and microfilament polymerization status [42]. The microtubule of eukaryotic cells is composed of α and β-Tubulin heterodimer [53]. Microtubule as the core element of cytoskeleton plays a key role in cell migration, mitosis, cell polarity, intracellular transport and cell morphogenesis [54, 55]. It undergoes depolymerization and rearrangement in the process of cell enlargement, which is a depolymerization status in the active meristem [56]. The abnormal expression of *α-Tubulin* gene can cause the disorder of microtubule structure in *Arabidopsis* root tip cells, which affects the normal cell division of root tips [57, 58]. The depolymerization of Actin protein in cytoskeleton assembly microfilaments can inhibit plant apical tissue growth, and this biological process is performed by Actin depolymerizing factor (ADF) [59]. Cytoskeleton depolymerization was also observed when JA promoted potato tuber cell enlargement [16], and this dynamic change was conducive to tuber cell enlargement [60, 61]. The finding here that α-Tubulin (M0ZYR0), β-Tubulin (B5M4B1) and ADF (M0ZV99) were significantly down-regulated (*p* < 0.05) under 50 μM JA treatment. It was suggested that the high JA concentration might restrict the dynamic structure changes of cytoskeleton by inhibiting the expression of cytoskeleton-related proteins, which was not conducive to tuber cell enlargement during tuber development.

### The primary carbon metabolism was remodeled by JA to provide metabolism intermediates and energy for tuber development

**Fig. 10:**
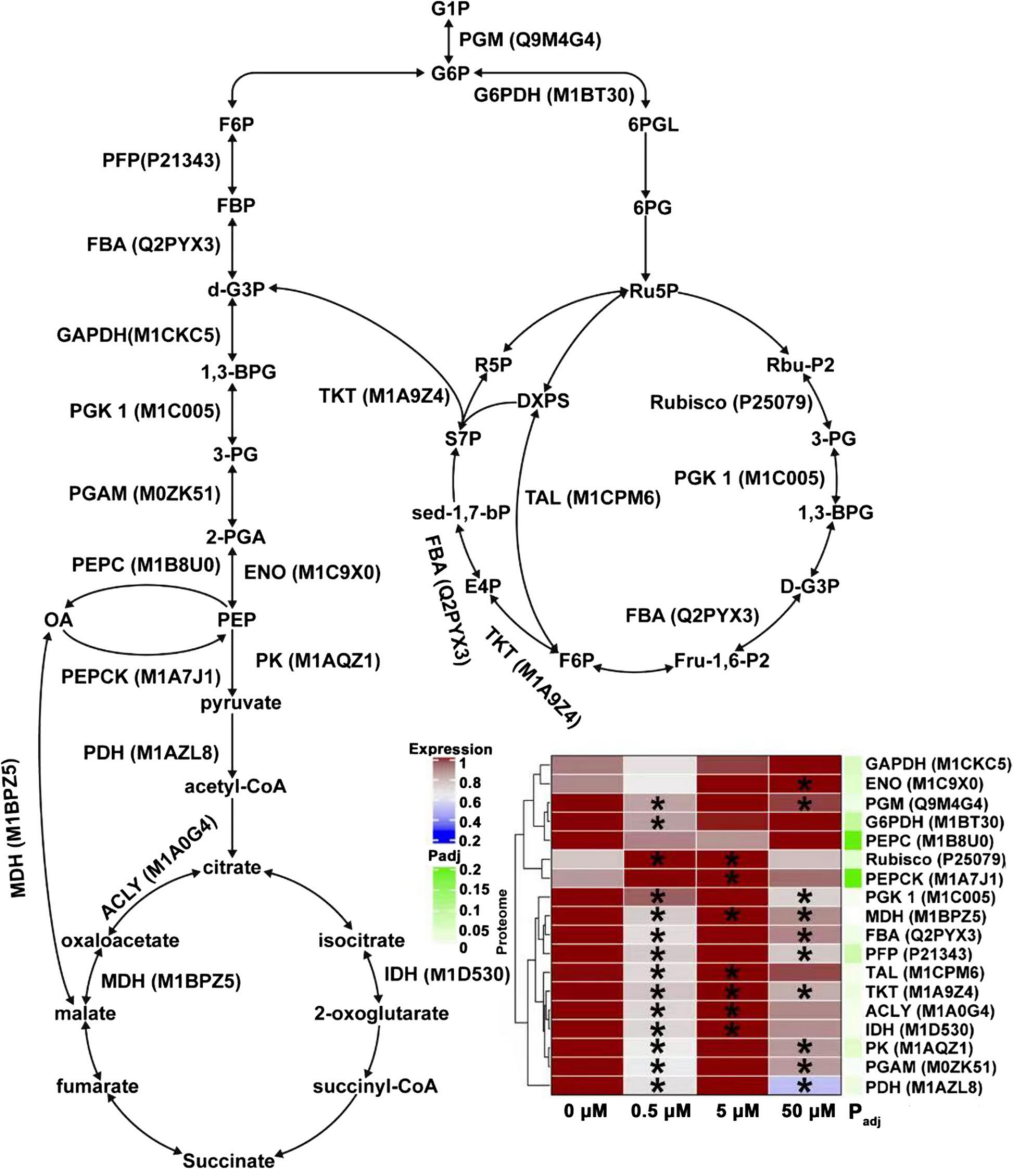
The JA-responsive DEPs involved in primary carbon metabolism during tuber development *in vitro*. The heat-map presented the expression change of these DEPs. The significance of *t*-test was presented by “*” (*p* < 0.05). The green band indicated the corrected *p*-value (P_adj_ < 0.05, one-way ANOVA analysis of variance followed by Bonferroni correction for multiple comparison) was mapped as an annotation of heatmap. ACLY, ATP citrate synthase; ENO, phosphopyruvate hydratase; FBA, fructose-bisphosphate aldolase; G6PDH, glucose-6-phosphate dehydrogenase; GAPDH, glyceraldehyde-3-phosphate dehydrogenase; IDH, isocitrate dehydrogenase; MDH, malate dehydrogenase; PDH, pyruvate dehydrogenase; PEPC, Phosphoenolpyruvate carboxylase; PEPCK, phosphoenolpyruvate carboxykinase; PFP, pyrophosphate: fructose 6-phosphate phosphotransferase; PGAM, phosphoglycerate mutase; PGK1, phosphoglycerate kinase 1; PGM, phosphoglucomutase; PK, pyruvate kinase; Rubisco, ribulose 1,5-bisphosphate carboxylase; TAL, transaldolase; TKT, transketolase

The primary carbon metabolism can produce precursors required for secondary metabolism and generate energy, which makes the fixed carbon flow between energy storage and consumption, thus affecting plant growth and development [62]. Some primary carbon metabolism-related enzymes were identified during potato tuber development regulated by JA. The expression pattern changes of these proteins were suggested that the primary carbon metabolism were remodeled by JA to meet the increased substance and energy requirement for tuber development (Fig. 10). Glycolysis is the core of bioenergy conversion that provides carbon skeleton for various metabolite biosynthesis [63]. Pyrophosphate: fructose 6-phosphate phosphotransferase (PFP) is the rate limiting enzyme in glycolysis pathway, which catalyzes the reversible transformation between fructose 6-phosphate (F6P) and fructose 1,6-diphosphate (FBP) [64]. Previous study has shown that the down-regulation of PFP expression leads to the increase of hexose phosphate pool in potato tubers, thus weakening starch synthesis [65]. Pyruvate kinase (PK) catalyzes the final reaction of glycolysis pathway to produce pyruvate that enters mitochondria as a substrate for respiration [66]. The decreased PK activity in potato tubers leads to a decrease of pyruvate and organic acid levels in tricarboxylic acid (TCA) cycle, accompanying by the carbon source distribution between glycolysis and starch [67]. The finding here that PFP (P21343) and PK (M1AQZ1) were significantly down-regulated (*p* < 0.05) under 0.5 and 50 μM JA treatments, which was suggested that the sufficient carbon source and energy cannot be supply for tuber development due to the decreased glycolysis rate. Pyruvate dehydrogenase (PDH) as a key enzyme in TCA cycle catalyzes the oxidative decarboxylation of pyruvate to form acetyl CoA [68]. Previous study has shown that the down-regulation of PDH leads to the abnormal development of tobacco flowers [69]. ATP citrate synthase (ACLY) catalyzes the conversion of acetyl CoA to citric acid in TCA cycle, and plays an important role in flower development, nutrient absorption and carbon skeleton source for nitrogen assimilation [70]. Isocitrate dehydrogenase (IDH) is considered to be a key regulatory node in TCA cycle, and plays important roles in maintaining 2-oxoglutarate level and regulating nitrogen assimilation [63]. The ACLY (M1A0G4) and IDH (M1D530) were found to be significantly down-regulated (*p* < 0.05) under 0.5 μM JA treatments, slightly down-regulated under 50 μM JA treatment, whereas significantly up-regulated (*p* < 0.05) under 5 μM JA treatment. The PDH (M1AZL8) also showed a significantly down-regulation expression (*p* < 0.05) under 0.5 and 50 μM JA treatment. It appeared that a certain JA concentration might accelerate TCA cycle to provide sufficient energy and substance for tuber development.

Several enzymes involved in pentose phosphate pathway and Calvin cycle were also identified in the present study. Transaldolase (TAL) and transketolase (TKT) are two key enzymes for substance and energy flow between glycolysis and pentose phosphate pathway [71]. Glucose-6-phosphate dehydrogenase (G6PDH) oxidizes glucose-6-phosphate (G6P) to produce 6-phosphogluconolactone and NADPH in pentose phosphate pathway, maintaining NADPH/NADP^+^ ratio in cells [72]. Ribulose 1,5-bisphosphate carboxylase (Rubisco) is one of the key enzymes in photosynthetic carbon assimilation [73]. The TAL (M1CPM6), TKT (M1A9Z4) and Rubisco (P25079) were found to be significantly up-regulated (*p* < 0.05) under 5 μM JA treatment, and the G6PDH (M1BT30) showed a slight up-regulation. It might increase the carbon inflow of other metabolic pathways from pentose phosphate pathway and Calvin cycle, thus resulting in the increase of potato tuber biomass during tuber development. Phosphoglucomutase (PGM) catalyzes the conversion between glucose-6-phosphate (G6P) and glucose-1-phosphate (G1P), which links Calvin cycle, starch metabolism and sucrose metabolism [74]. Previous study has shown that the down-regulation of PGM expression reduces potato tuber number and yield [75]. In this study, the PGM (Q4M4G4) was significantly down-regulated (*p* < 0.05) under 0.5 and 50 μM JA treatments, which might hinder the supply of glucose-1-phosphate (G1P) for starch synthesis, thus inhibiting tuber development.

### The reprogramming of protein biosynthesis, degradation and assembly was required for tuber development regulated by JA

**Fig. 11:**
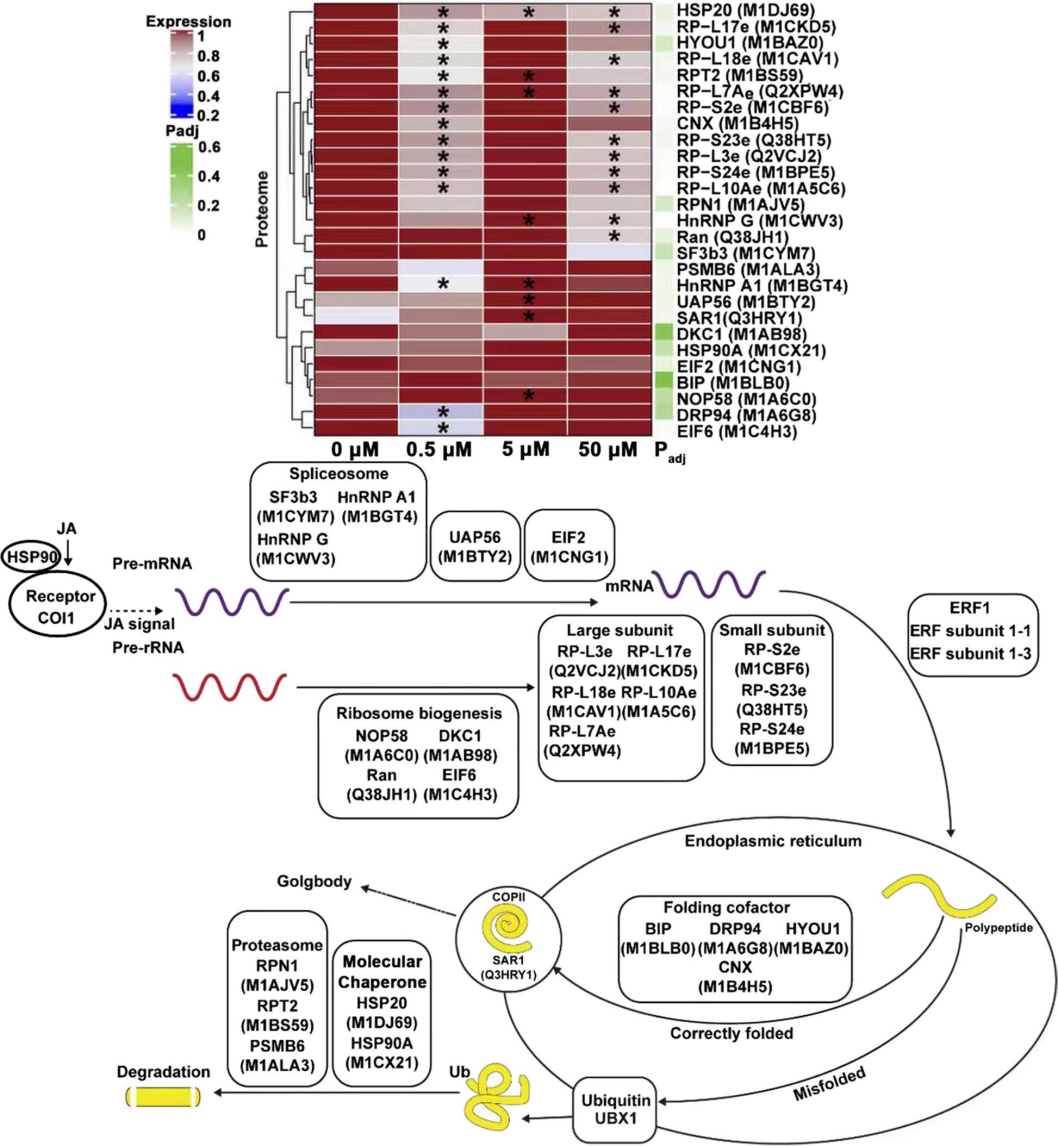
The JA-responsive DEPs involved in protein biosynthesis, degradation and assembly during tuber development *in vitro*. The heat-map presented the expression change of these DEPs. The significance of *t*-test was presented by “*” (*p* < 0.05). The green band indicated the corrected *p*-value (P_adj_ < 0.05, one-way ANOVA analysis of variance followed by Bonferroni correction for multiple comparison) was mapped as an annotation of heatmap. BIP, luminal binding protein; CNX, calnexin; GRP94, glucose-regulated protein 94; EIF2, eukaryotic translation initiation factor 2; EIF6, eukaryotic translation initiation factor 6; HnRNP A1, heterogeneous nuclear ribonucleoprotein A1; HnRNP G, heterogeneous nuclear ribonucleoprotein G; HSP20, heat shock protein 20; HSP90A, heat shock protein 90A; HYOU1, hypoxia up-regulated protein 1; NOP58, MAR-binding protein NOP58; PSMB6, proteasome subunit β type 6; RAN, GTP-binding nuclear protein; RPN1, proteasome subunit RPN1; RPT2, proteasome subunit RPT2; RP-L3e, ribosomal protein L3e; RP-L7Ae, ribosomal protein L7Ae; RP-L10Ae, ribosomal protein L10Ae; RP-L17e, ribosomal protein L17e; RP-L18e, ribosomal protein L18e; RP-S2e, ribosomal protein S2e; RP-S23e, ribosomal protein S23e; RP-S24e, ribosomal protein S24e; SF3b3, splicing factor 3b subunit 3; UAP56, DEAD-box ATP-dependent RNA helicases; SAR1, secretion-associated RAS superfamily 1; DKC1, Dyskerin 1

The precise regulation of protein metabolism plays crucial roles in numerous developmental and physiological processes in plants. Recent studies have established a certain connection between some key genes participating in various steps of cellular protein metabolism and phytohormone signal transduction [30, 76]. A large number of protein metabolism-related proteins were also identified in the present study, which was mainly involved in pre-mRNA processing and translation, protein biosynthesis, degradation and assembly (Fig. 11). It appeared that the reprogramming of protein biosynthesis, degradation and assembly might be required for potato tuber development regulated by JA. RNA splicing is an essential process to produce mature mRNA in eukaryotes [77]. Heterogeneous nuclear ribonucleoproteins (HnRNPs) as a large family of RNA binding proteins play multiple roles in pre-mRNA splicing, transcription, translation and turnover [78, 79], which are involved in the regulation of flower development, circadian rhythms, hormone signaling, stress response and phloem transport in plants [80–82]. DEAD-box ATP-dependent RNA helicases is responsible for the entry of nuclear pre-mRNA into cytoplasm for splicing and pre-translational processing [83]. MAR-binding protein participates in the composition of dense fibrous complex (DFC) in plant nucleolus, and the DFC contains factors are involved in pre-RNA processing [84]. The HnRNP G (M1CWV3), HnRNP A1 (M1BGT4) and DEAD-box ATP-dependent RNA helicases UAP56 (M1BTY2), MAR-binding protein NOP58 (M1A6C0) were found to be significantly up-regulated (*p* < 0.05) under 5 μM JA treatment, which might promote protein translation by improving the efficiency of pre-mRNA splicing and mRNA transport during tuber development.

The protein biosynthesis and transport is one of the key determinants for the rapid division of cells [85]. Ribosomal proteins (RPs) are a kind of highly conserved proteins that make up ribosomal subunits involved in the cellular process of translation [86]. The expression of RPs is higher in rapidly dividing cells, which can be stimulated by environment and growth factors [87, 88]. Knockout of *AtRPL23aA* resulted in the slow growth and reduced fertility of *Arabidopsis* plants [89]. In this study, several RPs including RP large subunit (Q2VCJ2, M1CDK5, M1CAV1, M1A5C6 and Q2XPW4) and RP small subunit (M1CBF6, Q38HT5 and M1BPE5) were significantly down-regulated (*p* < 0.05) under 0.5 and 50 μM JA treatments. It might hinder mRNA/ribosome interactions early in translation, which was not conducive to tuber development. Ran is a kind of small GTPase that plays a pivotal role in mitotic spindle assembly, nuclear envelope assembly and protein transport from nucleus to cytoplasm [90] Overexpression of *TaRAN1* in *Arabidopsis* and rice can increase the proportion of cells in G2 phase of cell cycle, and lead to an elevated mitotic index and prolonged life cycle [91]. Ran (Q38JH1) was found to be significantly down-regulated (*p* < 0.05) under 5 μM JA treatment, which might promote tuber development by accelerating protein transport and promoting tuber cell division. Secretion-associated RAS superfamily (SAR) is involved in protein transport from endoplasmic reticulum (ER) to Golgi apparatus by a coat protein complex II (COPII)-mediated pathway [92]. Simultaneous knockdown of *OsSar1* in rice endosperm can prevent the transport of glutenin and α-globulin from ER to Golgi apparatus, resulting in floury and shrunken seeds [93]. SAR1 (Q3HRY1) was also found to be significantly down-regulated (*p* < 0.05) under 5 μM JA treatment, which might promote tuber storage proteins exiting from ER and accumulating during tuber development.

Proteolysis is necessary for the removal of abnormal, modified and mistargeted proteins, and altering the balance of proteins [94]. Several proteasome components including RPN1 (M1AJV5), RPT2 (M1BS59) and PSMB6 (M1ALA3) were found to be significantly down-regulated (*p* < 0.05) under 5 μM JA treatment, which might play key roles in maintaining strict protein quality control and degrading specific sets of proteins during tuber development. Chaperones are responsible for protein folding, assembly, translocation and degradation in many normal cellular processes [95]. Calnexin (CNX) acts as a molecular chaperone that plays multiple roles in Ca^2+^ binding, lectin-like activity, and recognition and degradation of misfolded proteins [96]. DRP94 is a member of HSP90 protein family in ER that is involved in the regulation of Ca^2+^ in cells, thus promoting the transport of endoplasmic reticulum proteins [97]. HYOU1 is a homologous protein of HSP70 family of chaperone proteins in ER that assists in protein folding, assembly and disassembly of protein complexes, pulling polypeptides from ribosomes and transmembrane pores, inactivating signaling proteins and controlling their degradation [98]. The CNX (M1B4H5), DRP94 (M1A6G8) and HYOU1 (M1BAZ0) were found to be significantly down-regulated (*p* < 0.05) under 0.5 μM JA treatment, which might reduce the degradation ability of denatured proteins and the transport of newly synthesized peptides during tuber development.

## Conclusion

The present study first integrated physiological and proteomic analysis to investigate the molecular events of potato tuber development *in vitro* regulated by exogenous JA. The DEPs that play a variety of cellular functions were identified by iTRAQ-based proteomic analysis, which were involved in a dynamic network for tuber development. It indicated that the promotion effects of low JA concentration (especially 5 μM JA) on tuber development mainly exhibited in three major cellular processes (Figs. 9, 10 and 11). First, low JA concentration might promote tuber cell expansion by regulating the expression of cell wall polysaccharide synthesis and cytoskeleton formation-related proteins. Second, low JA concentration might cause the remodeling of carbon source distribution and energy flow to provide metabolism intermediates and energy for tuber development by regulating the expression of primary carbon metabolism-related enzymes. Third, low JA concentration might cause the reprogramming of protein biosynthesis, degradation and assembly to promote tuber protein biosynthesis and maintain strict protein quality control during tuber development. This study provided a comprehensive overview on the functional protein profile changes of tuber development regulated by JA.

## Supplementary Information

**Additional file 1: Table S1.** Proteins quantified by iTRAQ during tuber development regulated by JA *in vitro*.

**Additional file 2: Table S2.** The identification of credible proteins appearing simultaneously in three biological replicates.

**Additional file 3: Table S3.** The differential expression changes (Fold change ≥ 1.2 or ≤ 0.83, p < 0.05) of DEPs in each group (0.5 μM JA / control, 5 μM JA / control, 50 μM JA / control, 5 μM JA / 0.5 μM JA, 50 μM JA / 0.5 μM JA, 50 μM JA / 5 μM JA).

**Additional file 4: Table S4.** The protein name and information of JA-responsive DEPs presented in the heatmap of Fig. 4B.

**Additional file 5: Fig. S1. **The original gels for COI1, HSP90 and LOX2 immunoblot analysis.

**Additional file 6: Fig. S2. **The original immunoblot images of COI1. In order to save antibody and chromogenic reagent, the membranes were cut into strips just 1 cm above and below the 70 KDa molecular weight marker after transfer to a PVDF membrane and used for COI1 immunoblot.

**Additional file 7: Fig. S3. **The original immunoblot images of HSP90. In order to save antibody and chromogenic reagent, the membranes were cut into strips just between the 70 KDa and 100 KDa molecular weight markers after transfer to a PVDF membrane and used for HSP90 immunoblot.

**Additional file 8: Fig. S4. **The original immunoblot images of LOX2. In order to save antibody and chromogenic reagent, the membranes were cut into strips just 1 cm above and below the 70 KDa molecular weight marker after transfer to a PVDF membrane and used for LOX2 immunoblot.

## Data Availability

The raw data presented in this study are available on request from the corresponding author. The data are not yet publicly available since the project is still ongoing.
