# Supplementary figures and images for: Physiological and protein profiling analysis provides insight into the underlying molecular mechanism of potato tuber development regulated by jasmonic acid in vitro
Source: BMC Plant Biol. 2022 Oct 10;22:481. doi: 10.1186/s12870-022-03852-x (PMC9549635; doi:10.1186/s12870-022-03852-x)

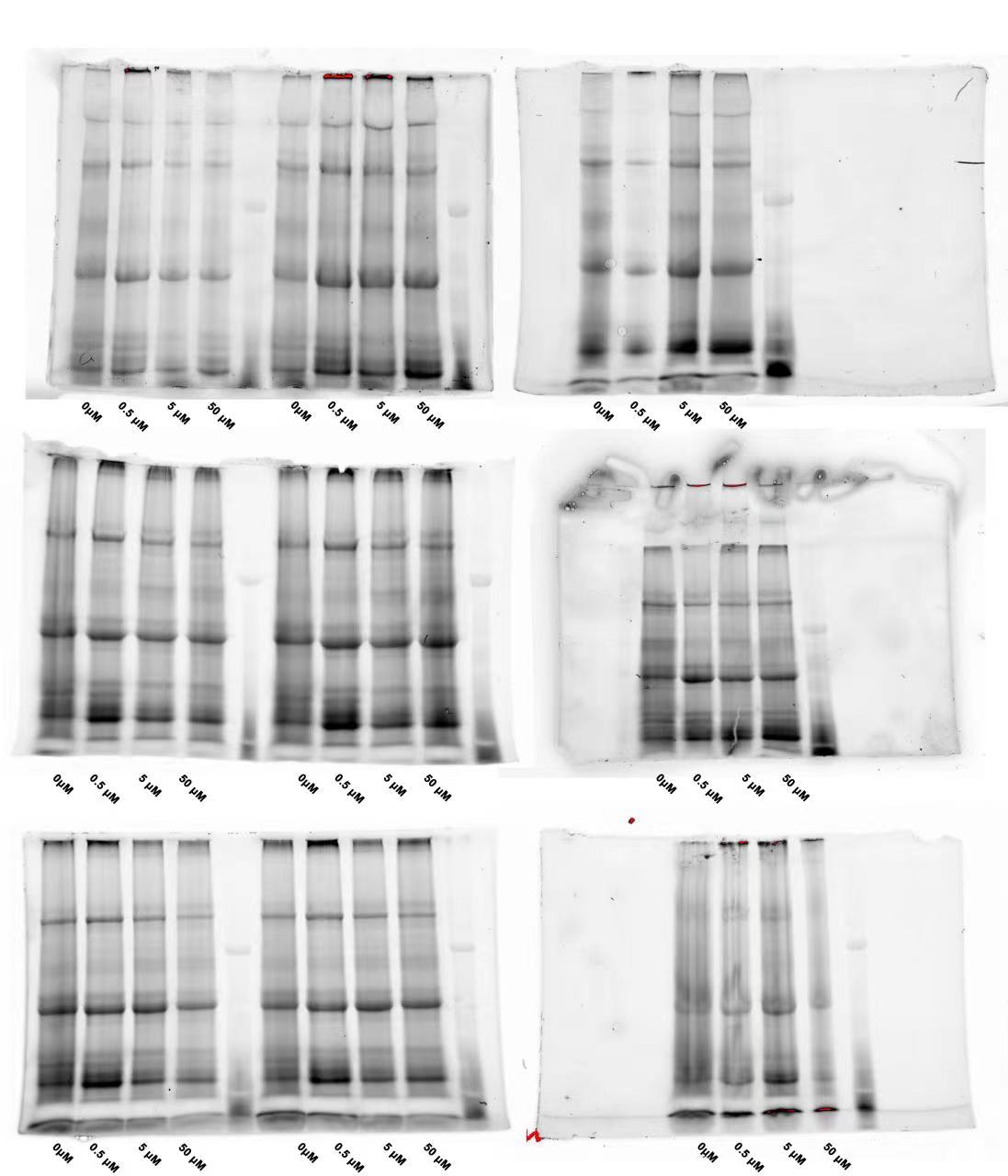

Supplement: Supplementary file 5 — Additional file 5: Fig. S1. The original gels for COI1, HSP90 and LOX2 immunoblot analysis. [file 12870_2022_3852_MOESM5_ESM.jpg]

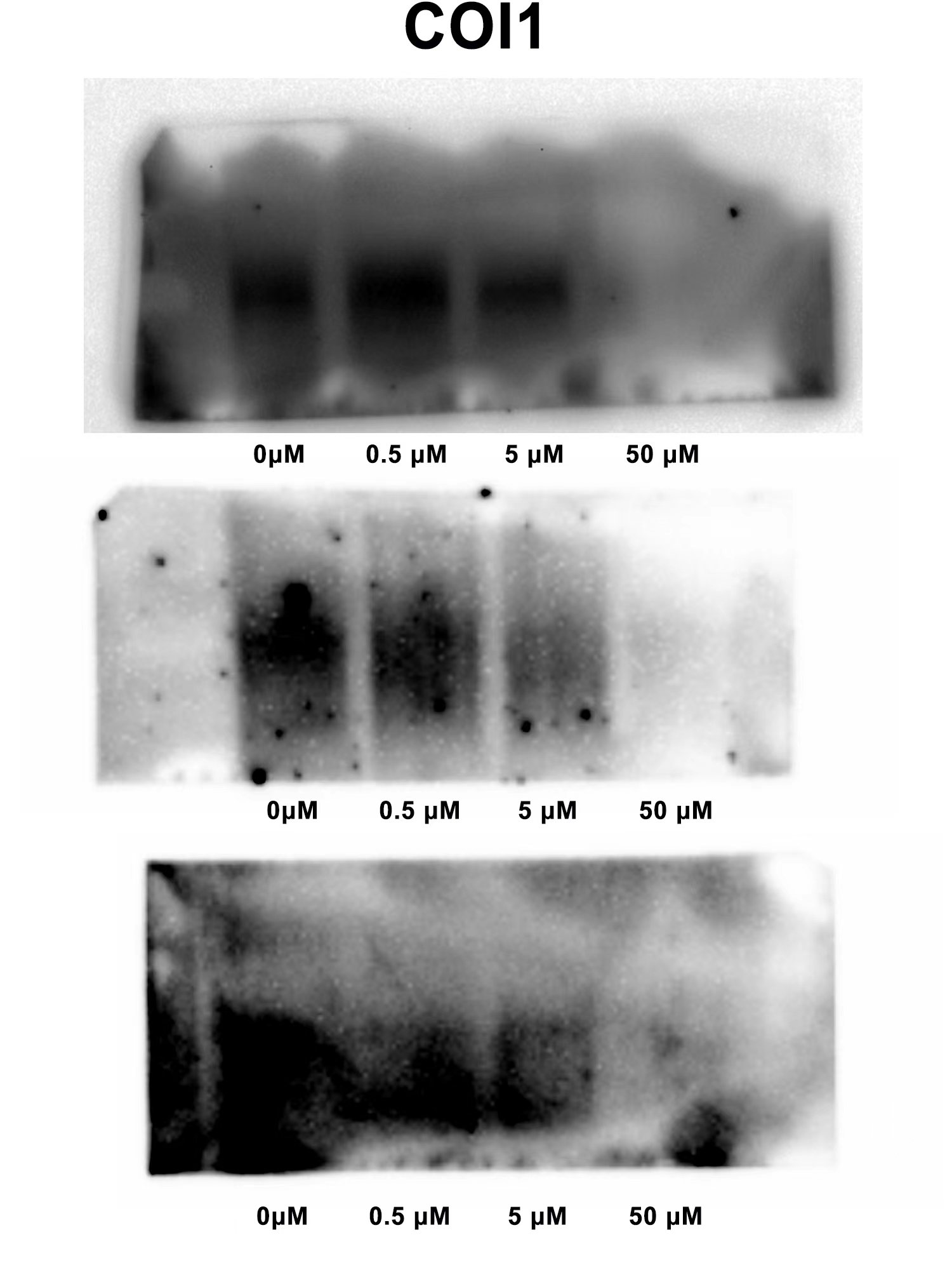

Supplement: Supplementary file 6 — Additional file 6: Fig. S2. The original immunoblot images of COI1. In order to save antibody and chromogenic reagent, the membranes were cut into strips just 1 cm above and below the 70 KDa molecular weight marker after transfer to a PVDF membrane and used for COI1 immunoblot. [file 12870_2022_3852_MOESM6_ESM.jpg]

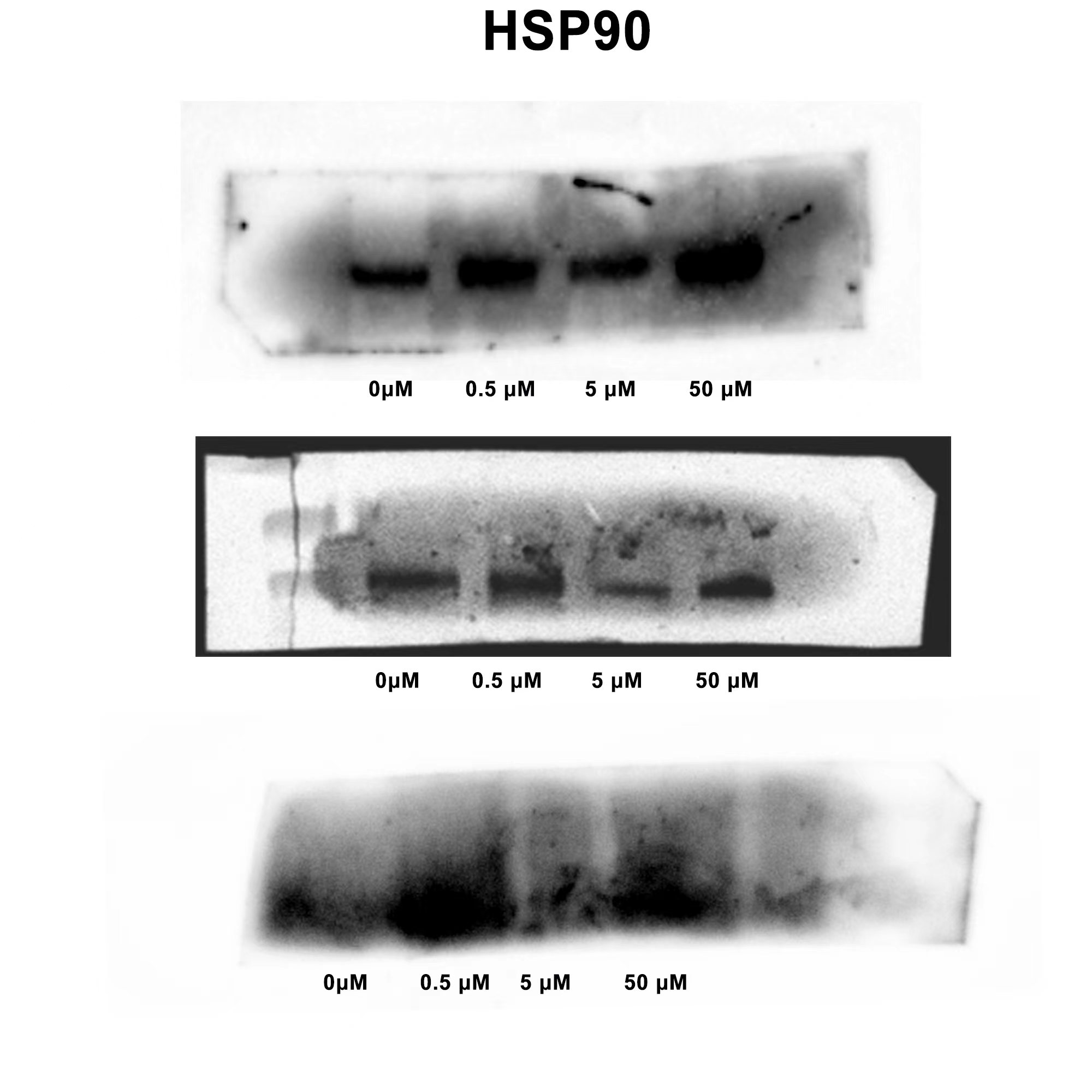

Supplement: Supplementary file 7 — Additional file 7: Fig. S3. The original immunoblot images of HSP90. In order to save antibody and chromogenic reagent, the membranes were cut into strips just between the 70 KDa and 100 KDa molecular weight markers after transfer to a PVDF membrane and used for HSP90 immunoblot. [file 12870_2022_3852_MOESM7_ESM.jpg]

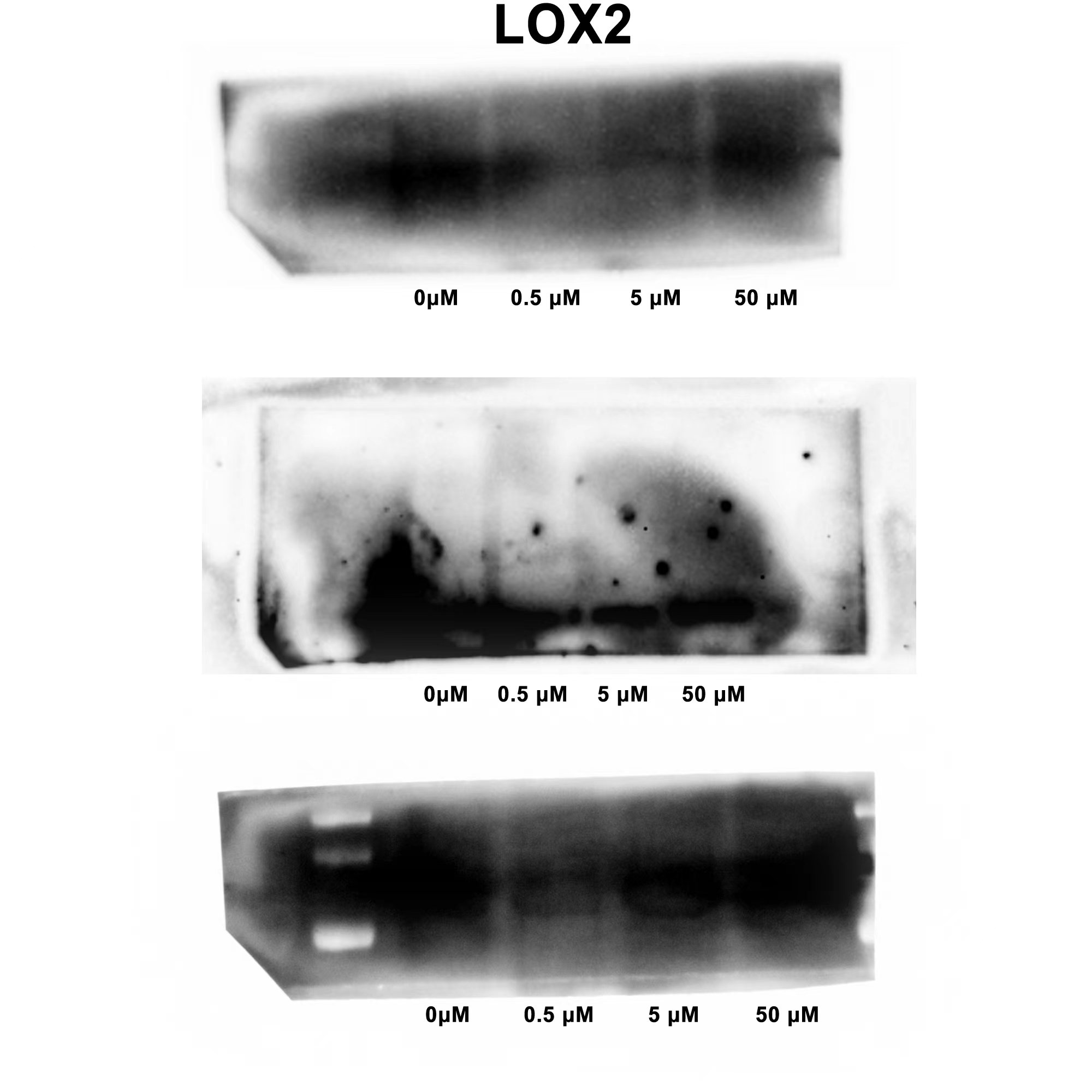

Supplement: Supplementary file 8 — Additional file 8: Fig. S4. The original immunoblot images of LOX2. In order to save antibody and chromogenic reagent, the membranes were cut into strips just 1 cm above and below the 70 KDa molecular weight marker after transfer to a PVDF membrane and used for LOX2 immunoblot. [file 12870_2022_3852_MOESM8_ESM.jpg]
